# Supplementary material for: Prehospital delay is an important risk factor for mortality in community-acquired bloodstream infection (CA-BSI): a matched case–control study
Source: BMJ Open. 2021 Nov 17;11(11):e052582. doi: 10.1136/bmjopen-2021-052582 (PMC8603295; doi:10.1136/bmjopen-2021-052582)
Supplement: Supplementary data [file bmjopen-2021-052582supp001.pdf]

Supplementary Table 1. Charlson Comorbidity Index

Comorbid Condition	ICD-9-CM Diagnosis Codes	ICD-10-CA Diagnosis Codes	Weight	Updated Weight
**Myocardial Infarction**	410, 412	I21, I22, I25.2	1	0
**Congestive Heart Failure**	398.91, 402.01, 402.11, 402.91, 404.01, 404.03, 404.11, 404.13, 404.91, 404.93, 425.4–425.9, 428 (hosp), 398, 402, 425, 428 (med)	I09.9, I11.0, I13.0, I13.2, I25.5, I42.0, I42.5–I42.9,I43, I50, P29.0	1	2
**Peripheral Vascular Disease**	093.0, 437.3, 440, 441, 443.1–443.9, 447.1, 557.1, 557.9, V43.3 (hosp) 440, 441, 443, 447, 557 (med)	I70, I71, I73.1, I73.8, I73.9, I77.1, I79.0, I79.2, K55.1, K55.8, K55.9, Z95.8, Z95.9	1	0
**Cerebrovascular Disease**	362.34, 430–438 (hosp) 430–438 (med)	G45, G46, H34.0, I60–I69	1	0
**Dementia**	290, 294.1, 331.2 (hosp) 290, 294, 331 (med)	F00–F03, F05.1, G30, G31.1	1	2
**Chronic Pulmonary Disease**	416.8, 416.9, 490–505, 506.4, 508.1, 508.8 (hosp), 416, 490–496, 500–505 (med)	I27.8, I27.9,J40–J47, J60–J67 J68.4, J70.1, J70.3	1	1
**Connective Tissue Disease-Rheumatic Disease**	446.5, 710.0–710.4, 714.0–714.2, 714.8, 725 (hosp), 446, 710, 714, 725 (med)	M05, M06, M31.5, M32–M34, M35.1, M35.3, M36.0	1	1
**Peptic Ulcer**	531–534	K25–K28	1	0
**Mild Liver Disease**	070.22, 070.23, 070.32, 070.33, 070.44, 070.54, 070.6, 070.9, 570, 571, 573.3, 573.4, 573.8, 573.9, V42.7 (hosp) 070, 570, 571, 573 (med)	B18, K70.0–K70.3, K70.9, K71.3–K71.5, K71.7, K73, K74, K76.0, K76.2–K76.4, K76.8, K76.9, Z94.4	1	2
**Diabetes without Chronic Complications**	250.0–250.3, 250.8, 250.9 (hosp) 250 (med)	E10.0, E10.1, E10.6, E10.8, E10.9, E11.0, E11.1, E11.6, E11.8, E11.9, E12.0, E12.1, E12.6, E12.8, E12.9, E13.0, E13.1, E13.6, E13.8, E13.9, E14.0, E14.1, E14.6, E14.8, E14.9	1	0
**Diabetes with Chronic Complications**	250.4–250.7 (med n/a)	E10.2–E10.5, E10.7, E11.2–E11.5, E11.7, E12.2–E12.5, E12.7, E13.2–E13.5, E13.7, E14.2–E14.5, E14.7	2	1
**Paraplegia or Hemiplegia**	334.1, 342, 343, 344.0–344.6, 344.9 (hosp), 334, 342–344 (med)	G04.1, G11.4, G80.1, G80.2, G81, G82, G83.0–G83.4, G83.9	2	2

**Renal Disease**	403.01, 403.11, 403.91, 404.02, 404.03, 404.12, 404.13, 404.92, 404.93, 582, 583.0–583.7, 585, 586, 588.0. V42.0, V45.1, V56 (hosp), 403, 582, 583, 585, 586, 588, V56 (med)	I12.0, I13.1, N03.2–N03.7, N052–N05.7, N18, N19, N25.0, Z49.0–Z49.2, Z94.0, Z99.2	2	1
**Cancer ***	140–172, 174–195.8, 200–208, 238.6 (hosp), 140–172, 174–195, 200–208, 238 (med)	C00–C26, C30–C34, C37–C41, C43, C45–C58, C60–C76, C81–C85, C88, C90–C97	2	2
**Moderate or Severe Liver Disease**	456.0–456.2, 572.2–572.4, 572.8 (hosp), 456, 572 (med)	I85.0, I85.9, I86.4, I98.2, K70.4, K71.1, K72.1, K72.9, K76.5–K76.7,	3	4
**Metastatic Carcinoma****	196–199	C77–C80	6	6
**HIV/AIDS**	042–044	B20–B22, B24	6	4

*Abbreviations: AIDS, acquired immunodeficiency syndrome; HIV, human immunodeficiency virus. * Including lymphoma and leukemia, except malignant neoplasm of skin ** or Metastatic solid tumour.*

Supplementary Table 2. Distribution of comorbidities

	**Non-survivors n=195**	**Survivors n=195**	**p-value**
**Comorbidity (%)**			
Myocardial infarction	31 (16)	41 (21)	0.19
Congestive heart failure	56 (29)	50 (26)	0.50
Peripheral vascular disease	14 (7)	16 (8)	0.70
Cerebrovascular disease	30 (15)	28 (14)	0.78
Dementia	15 (8)	11 (6)	0.42
Chronic pulmonary disease	35 (18)	33 (17)	0.79
Connective Tissue Disease-Rheumatic Disease	13 (7)	22 (11)	0.11
Peptic ulcer disease	3 (2)	3 (2)	>0.99
Mild liver disease	8 (4)	9 (5)	0.80
Diabetes without chronic complications	43 (22)	62 (32)	*0.03*
Diabetes with chronic complications	8 (4)	9 (5)	0.80
Paraplegia and hemiplegia	13 (7)	11 (6)	0.67
Renal disease	34 (17)	25 (13)	0.20
Cancer	67 (34)	44 (23)	*0.01*
Moderate or severe liver disease	9 (5)	3 (2)	0.08
Metastatic carcinoma	28 (14)	14 (7)	*0.02*
HIV/AIDS	0	0	-
Charlson (update weight) (SD)	3.2 (3.0)	2.3 (2.4)	*0.01*

Data are presented as no. (%) or mean (SD). Pearson chi2, Fisher's exact test or T-test, as appropriate.

Supplementary Table 3. Distribution of primary pathogen*

**Gram negative**	**Non-survivors n=195**	**Survivors n=195**	**%**
**Enterobacterales**			
Escherichia coli	41	41	21
Klebsiella pneumoniae	14	14	7
Klebsiella oxytoca	3	3	2
Enterobacter cloacae	2	2	1
Proteus Mirabilis	4	4	2
Haemophilus influenzae	4	4	2
**Other than Enterobacterales**			
Pseudomonas aeruginosa	7	7	4
**Others**	7	7	4
**Gram positives**			
**Enterococci**			
Enterococcus faecalis	6	6	3
Enterococcus faecium	5	5	3
**Staphylococci**			
Staphylococcus aureus	52	52	27
Staphylococcus spp	5	5	3
**Streptococci**			
Streptococcus pneumoniae	16	16	8
Streptococcus pyogenes (A)	1	1	1
Streptococcus agalactiae (B)	4	4	2
Streptococcus group C,G	5	5	3
Streptococcus spp	11	11	6
***Candida* spp**	4	4	2
**Others**	4	4	2
**Polymicrobial-BSI**	9	8	4

**Primary pathogen reported in the table. In Polymicrobial-BSI, an expert group (physicians specialised in infectious diseases) evaluated which pathogen was primary.*

Supplementary Table 4. Hospital data - Triage at hospital (RETTS and NEWS 2) and contact cause at hospital

	**Non-survivors n=195**	**Survivors n=195**	**p-value**
**Vital signs (SD)***			
Body temp (BT)	37.6 (1.3) n189	38.2 (1.1) n194	*<0.01*
Systolic blood pressure (SBP)	112 (28) n190	131 (28) n193	*<0.01*
Diastolic blood pressure (DBP)	65 (18) n188	72 (17) n192	*<0.01*
Pulse rate (HR)	102 (26) n190	97 (22) n192	*0.04*
Respiratory rate (RR)	27 (10) n184	23 (7) n189	*<0.01*
Blood O_2 saturation (SPO_2)	92 (9) n187	95 (4) n192	*<0.01*
PaO_2/FiO_2	42 (21) n183	51 (20) n187	*<0.01*
Reaction Level Scale (RLS)	1.4 (0.9) n187	1.1 (0.46) n192	*<0.01*
**Laboratory median (Q1-Q3)**			
C-reactive protein (mg/L)	180 (87-304) n195	138 (50-225) n192	*<0.01*
White blood cell count ($x10^9$/L)	13 (8-19) n195	13(10-17) n191	0.42
Platelet count ($x10^9$/L)	194 (130-292) n195	212 (156-280) n191	0.09
Creatinine (µmol/L)	143 (94-216) n191	98 (74-139) n190	*<0.01*
eGFR MDRD (mL/min/1.73 m^2)	36 (22-58) n186	58 (35-74) n186	*<0.01*
Albumin (g/L)	23 (20-28) n109	29 (25-32) n100	*<0.01*
B-Erythrocytes ($x10^{12}$/L)	4 (3-4) n195	4 (4-5) n191	*0.04*
B-MCV (fL)	95 (91-101) n195	93 (90-97) n191	*<0.01*
Haemoglobin (g/L)	116 (101-136) n195	121 (109-138) n191	*0.02*
Procalcitonin (µg/L)	10 (2-54) n34	4 (1-31) n26	0.33
**Primary focus of infection (%)**			
Respiratory tract	39 (20)	46 (24)	0.39
Urinary tract	42 (22)	50 (26)	0.34
Skin and soft tissue	19 (10)	20 (10)	0.86
Abdomen	12 (6)	18 (9)	0.25
CNS	-	2 (1)	0.50
Indwelling catheter	1 (1)	0	>0.99
Endocarditis	1 (1)	1 (1)	>0.99
Unknown	46 (24)	43 (22)	0.72
Missing data	35 (18)	15 (8)	
**Triage**			
RETTS Red (%)	76 (46) n164	62 (35) n177	*0.03*
RETTS Orange (%)	51 (31) n164	71 (40) n177	0.08
RETTS Yellow (%)	32 (20) n164	42 (24) n177	0.35
RETTS Green (%)	5 (3) n164	2 (1) n177	0.27
RETTS Red plus Orange (%)	127 (77) n164	133 (75) n177	0.62
NEWS 2 score (SD)	7.1 (4.0) n163	5.0 (3.4) n181	*<0.01*

NEWS 2 score ≥5 (%)	114 (70) n163	89 (49) n181	*<0.01*
**Reason for first hospital contact (%)**			
Fever	86 (44)	119 (61)	*<0.01*
Chills	26 (13)	50 (26)	*0.02*
"Found on the floor"	18 (9)	5 (3)	*0.01*
Gastrointestinal symptoms	32 (16)	32 (16)	>0.99
Cough	8 (4)	15 (8)	0.13
Dyspnoea/breathing difficulties	60 (31)	46 (24)	0.11
Urinary tract symptoms	13 (7)	25 (13)	*0.04*
Rapid deterioration in general condition	81 (42)	48 (25)	*<0.01*
Fatigue	14 (7)	17 (9)	0.57

*Data are presented as no. (%) or mean (SD). Pearson chi2, Fisher's exact test or T-test, as appropriate. Laboratory data are calculated with median, interquartile 25th to 75th percentile range (Q1-Q3) and Mann–Whitney U. * Vital signs first registered at admission. RETTS: Red = life-threatening condition, Orange = potentially life-threatening. Yellow/Green = acute process can wait.*

Supplementary Table 5. Early hospital care - Time for ambulance transport and time to empirical antibiotic treatment based on triage score (RETTS)

Early hospital care	Non-survivors	Survivors	p-value
Paramedic alarm times, hours, median (Q1-Q3)	0,72 (0,57-0,95), n137	0,73 (0,58-0,98), n131	0.40
Time to empirical antibiotic treatment based on triage score (RETTS), hours, median (Q1-Q3):			
Red (triage 1)	1,52 (0,67-3,18), n68	1,62 (0,90-2,98), n62	0.50
Orange (triage 2)	2,63 (1,72-5,15), n50	3,07 (1,63-6,13), n69	0.67
Yellow (triage 3)	4,88 (3,72-7,30), n32	5,28 (2,75-9,97), n38)	0.80
Green (triage 4)*	9,67 (6,26), n5	11,23 (8,60), n2	0.79

*Data are presented as hours and median (Q1-Q3) or * mean and SD. Mann-Whitney U test or T-test are used, as appropriate. RETTS: Red = life-threatening condition, Orange = potentially life-threatening. Yellow/Green = acute process can wait.*

Supplementary Table 6. Distribution of Empirical antibiotics used

	Non-survivors (n186)	Survivors (n192)	p-value
**Classes of antibiotics:**			
Aminoglycosides	15 (8)	9 (5)	0.18
Penicillins + beta-lactamase inhibitors	53 (28)	43 (22)	0.18
Carbapenems	23 (12)	8 (4)	<0.01
Cephalosporins	90 (48)	121 (63)	<0.01
Quinolones	3 (2)	1 (1)	0.37
Trimethoprim-sulfamethoxazole	0 (0)	1 (1)	>0.99
Glycopeptides	1 (1)	1 (1)	>0.99
Lincosamides	9 (5)	5 (3)	0.25
Macrolides	3 (2)	2 (1)	0.68
Nitroimidazoles	3 (2)	2 (1)	0.68
Penicillins	15 (8)	20 (10)	0.53
Antifungals	1 (1)	2 (1)	>0.99
Others	1 (1)	0 (0)	0.49
**Monotherapy treatment**	156 (84)	173 (90)	0.08
**Combination therapy treatment**	31 (17)	21 (11)	0.11

Data are presented as no. (%). Pearson chi2 or Fisher's exact test as appropriate. Combination therapy treatment was defined as more than one agent given within 4 hours in order to broaden the antimicrobial spectrum. Seven patients received oral antibiotic treatment, 4 non-survivors and 3 survivors.
