# Supplementary material for: Prehospital delay is an important risk factor for mortality in community-acquired bloodstream infection (CA-BSI): a matched case–control study
Source: BMJ Open. 2021 Nov 17;11(11):e052582. doi: 10.1136/bmjopen-2021-052582 (PMC8603295; doi:10.1136/bmjopen-2021-052582)
Supplement: Author's manuscript [file bmjopen-2021-052582.draft_revisions.pdf]

BMJ Open

BMJ Open is committed to open peer review. As part of this commitment we make the peer review history of every article we publish publicly available.

When an article is published we post the peer reviewers' comments and the authors' responses online. We also post the versions of the paper that were used during peer review. These are the versions that the peer review comments apply to.

The versions of the paper that follow are the versions that were submitted during the peer review process. They are not the versions of record or the final published versions. They should not be cited or distributed as the published version of this manuscript.

BMJ Open is an open access journal and the full, final, typeset and author-corrected version of record of the manuscript is available on our site with no access controls, subscription charges or pay-per-view fees (http://bmjopen.bmj.com).

If you have any questions on BMJ Open's open peer review process please email info.bmjopen@bmj.com

BMJ Open

Prehospital delay is an important risk factor for mortality in community-onset bloodstream infection (CO-BSI): A matched case-control study

Journal:	*BMJ Open*
Manuscript ID	bmjopen-2021-052582
Article Type:	Original research
Date Submitted by the Author:	20-Apr-2021
Complete List of Authors:	Holmbom, Martin; Linköping University, Department of Biomedical and Clinical Sciences; Linköping University, Department of Urology<br>Andersson, Maria; Linköping University, Division of Infectious Diseases, Department of Biomedical and Clinical Sciences, Faculty of Medicine and Health Sciences, Linköping University, Linköping, Sweden<br>Berg, Sören; Unit for Cardiovascular Sciences, Faculty of Medicine and Health Sciences<br>Eklund, Dan; Linköping University, Division of Infectious Diseases, Department of Biomedical and Clinical Sciences, Faculty of Medicine and Health Sciences, Linköping University, Linköping, Sweden<br>Sobczynski, Pernilla; Linköping University, Division of Infectious Diseases, Department of Biomedical and Clinical Sciences, Faculty of Medicine and Health Sciences, Linköping University, Linköping, Sweden<br>Moberg, Anna; Linköping University, Department of Health, Medicine and Caring Sciences, Linkoping University, Linkoping, Sweden<br>Wilhelms, Daniel; Linköping University, Department of Health and Medical Sciences,; Local Health Care Services in Central Östergötland, Department of Emergency Medicine<br>Fredrikson, Mats; Department of Clinical and Experimental Medicine, Faculty of Health Sciences<br>Balkhed, Åse Östholm; Linköping University, Division of Infectious Diseases, Department of Biomedical and Clinical Sciences, Faculty of Medicine and Health Sciences, Linköping University, Linköping, Sweden<br>Hanberger, Håkan ; Linköping University
Keywords:	Epidemiology < INFECTIOUS DISEASES, Adult intensive & critical care < INTENSIVE & CRITICAL CARE, ACCIDENT & EMERGENCY MEDICINE

SCHOLARONE™
Manuscripts

I, the Submitting Author has the right to grant and does grant on behalf of all authors of the Work (as defined in the below author licence), an exclusive licence and/or a non-exclusive licence for contributions from authors who are: i) UK Crown employees; ii) where BMJ has agreed a CC-BY licence shall apply, and/or iii) in accordance with the terms applicable for US Federal Government officers or employees acting as part of their official duties; on a worldwide, perpetual, irrevocable, royalty-free basis to BMJ Publishing Group Ltd ("BMJ") its licensees and where the relevant Journal is co-owned by BMJ to the co-owners of the Journal, to publish the Work in this journal and any other BMJ products and to exploit all rights, as set out in our licence.

The Submitting Author accepts and understands that any supply made under these terms is made by BMJ to the Submitting Author unless you are acting as an employee on behalf of your employer or a postgraduate student of an affiliated institution which is paying any applicable article publishing charge ("APC") for Open Access articles. Where the Submitting Author wishes to make the Work available on an Open Access basis (and intends to pay the relevant APC), the terms of reuse of such Open Access shall be governed by a Creative Commons licence – details of these licences and which Creative Commons licence will apply to this Work are set out in our licence referred to above.

Other than as permitted in any relevant BMJ Author's Self Archiving Policies, I confirm this Work has not been accepted for publication elsewhere, is not being considered for publication elsewhere and does not duplicate material already published. I confirm all authors consent to publication of this Work and authorise the granting of this licence.

For peer review only

Prehospital delay is an important risk factor for mortality in community-onset bloodstream infection (CO-BSI): A matched case-control study

Martin Holmbom[1,2], Maria Andersson[1], Sören Berg[3], Dan Eklund[1], Pernilla Sobczynski[1], Daniel Wilhelms[4], Anna Moberg[5], Mats Fredrikson[6], Åse Östholm Balkhed[1*], Håkan Hanberger[1*]

1 Division of Infectious Diseases, Department of Biomedical and Clinical Sciences, Faculty of Medicine and Health Sciences, Linköping University, Linköping, Sweden, **2** Department of Urology and Department of Biomedical and Clinical Sciences, Linköping University, Linköping, Sweden, **3** Unit for Cardiovascular Sciences, Faculty of Medicine and Health Sciences, **4** Department of Emergency Medicine in Linköping, and Department of Biomedical and Clinical Sciences, Linköping University, Linköping, Sweden **5** Department of Health, Medicine and Caring Sciences, Linkoping University, Linkoping, Sweden, **6** Department of Biomedical and Clinical Sciences and Forum Östergötland, Faculty of Medicine and Health Sciences. * Contributing equally to this work.

Corresponding author: Martin Holmbom, martin.holmbom@regionostergotland.se

ABSTRACT

Objectives: The aim of this study was to identify prehospital and early hospital risk factors associated with 30-day mortality in patients with blood culture-confirmed community-onset bloodstream infection (CO-BSI) in Sweden.

Methods: A retrospective case-control study of 1,624 patients with CO-BSI (2015-2016), 195 non-survivors satisfying the inclusion criteria were matched 1:1 with 195 survivors for age, gender, and microorganism. All forms of contact with a healthcare provider for symptoms of infection within 7 days prior CO-BSI episode were registered. Logistic regression was used to analyse risk factors for 30-day all-cause mortality.

Results: Of the 390 patients, 61% (115 non-survivors and 121 survivors) sought prehospital contact. The median time from first prehospital contact till hospital admission was 13 hours (6-52) for non-survivors and 7 hours (3-24) for survivors (p<0.01). Several risk factors for 30-day all-cause mortality were identified: prehospital delay OR=1.26 (CI: 1.07-1.47), p<0.01; severity of illness (SOFA-score) OR=1.60 (CI: 1.40-1.83), p<0.01; comorbidity score (updated Charlson Index) OR=1.13 (CI: 1.05-1.22), p<0.01; and inadequate empirical antimicrobial therapy OR=3.92 (CI: 1.64-9.33), p<0.01. In a multivariable model, prehospital delay >24 hours from first contact

For peer review only

remained an important risk factor for 30-day all-cause mortality due to CO-BSI OR=6.17 (CI: 2.19-17.38), p<0.01.

Conclusion: Prehospital delay was the most important risk factor for 30-day all-cause mortality. Increased awareness and earlier detection of bloodstream infection in prehospital and early hospital care is critical for rapid initiation of adequate management and antibiotic treatment.

Article summary

Strengths and limitations of this study

- This study provides important results regarding mortality risk factors in prehospital and early hospital care in a well-defined case-control study of patients with community-onset bloodstream infection.
- Non-surviving patients were matched 1:1 with survivors for age, gender, and microorganism to evaluate risk factors for 30-day mortality.
- Data on limitation of level of care were collected and evaluated as a potential confounding factor.
- Only empirical antibiotic treatment was analysed, early changes in therapy were not considered.
- Data regarding patient delay *i.e.,* time from onset of symptoms to contact with prehospital or hospital care, were unavailable.

For peer review only

Background

Bloodstream infection (BSI) is a major cause of morbidity and mortality worldwide, with sepsis and septic shock as important complications [1-5]. In Europe, the number of BSIs and number of deaths due to BSI each year have been estimated to be 1.2 million and 157 000 respectively [6]. Most studies in Nordic countries report an increase in the incidence of BSI [7-10] and an increase in associated mortality [7, 9], but a low level of antibiotic resistance [11].

The increasing incidence of community-onset bloodstream infection (CO-BSI) has become a major problem. This may be due to an increase in healthcare-associated BSI related to complex medical care of aged patients with comorbidity in the community setting [7, 12]. CO-BSI and the risk of transition to sepsis and septic shock with life-threatening organ dysfunction, requires early identification for prompt initiation of life-saving measures and improvement in outcome [13-17]. There are many studies focusing on improving early detection of patients at risk for developing sepsis in the Emergency Department (ED) [18-22], but there are few studies on prehospital care prior to evaluation by paramedics or at the ED.

In most West European countries, prehospital healthcare is available at several levels. In this study, prehospital care is defined as healthcare contact prior to transport by ambulance or entering the Emergency Department (ED). In Sweden, first contact with the healthcare service is usually by a phone call to the primary healthcare center (PHCC) or to the National Healthcare Guide tel: 1177 (NHG). In urgent situations, the emergency medical service (*i.e.*, paramedic ambulance service) is contacted directly. PHCCs usually provide a call-back system where a nurse rings during office hours and assesses the need for clinical assessment. The NHG offers immediate medical advice over the phone twenty-four hours a day. It is manned by specially trained nurses using a decision-support algorithm, they assess the need for further care and provide advice and/or recommend other healthcare services.

For peer review only

Few studies have addressed prehospital care of sepsis prior to evaluation at the ED or by the paramedic [23, 24], and not one, to our knowledge, on the impact of delay in prehospital care on 30-day mortality in a well-defined case-control study on patients with CO-BSI. The aim of this study was to identify prehospital and early hospital risk factors associated with 30-day mortality in patients with culture-confirmed community-onset bloodstream infection.

METHODS

For peer review only

Study design and setting

This was a case-control study on risk factors for 30-day mortality in patients with CO-BSI. From a database of culture-confirmed CO-BSI in the Swedish county of Östergötland, samples of cases (non-survivors) and matched controls (survivors) over a two-year period (from 1st January 2015 until 31st December 2016) were selected. The county of Östergötland, with a population of 445,000 in December 2015 and 452,000 in December 2016, had four hospitals, one tertiary care university hospital, two general hospitals, one district hospital, 44 PHCCS and four out of hours centers.

Data collection

From the microbiology laboratory database, the following dataset was collected: blood culture results, number of aerobic and anaerobic blood culture vials taken, sites of puncture; species identification, and susceptibility patterns. The dataset was entered into a second database where it was linked to the patient-administration system providing the following data on all patients with a BSI episode: gender, age, comorbidity, admitting department, date of admission, date of discharge, and mortality.

A total of 2,356 BSI episodes were identified, of which 1,624 were CO-BSIs. To determine the impact of prehospital and early hospital care on 30-day all-cause mortality 2015-2016, we matched

a group of non-survivors (cases) with survivors (controls) that satisfied the following inclusion criteria: adult (≥ 18 years), culture-confirmed CO-BSI with a significant pathogen, registered in the County of Östergötland, treated in a hospital in the County of Östergötland, non-survivor - dead within 30 days from positive blood culture and survivor - alive at 30 days.

In all, 195 non-survivors met the inclusion criteria and were matched with 195 survivors for age (+/- 10 years), gender, and microorganisms (Fig 1).

Data on all infection-related contacts with a healthcare provider (telephone contact or physical contact) ≤7 days prior to a positive blood culture were collected and analysed. For each contact, the following were documented in the Case Report Form: profession of healthcare provider, suspected diagnosis, vital signs, primary focus of infection, and initial advice given. In the event of ambulance transport, information on time to reach the patient, time to reach ED, vital signs, intravenous fluid administration, and oxygen administration were extracted from the mission report.

The following data on management in the ED were collected: limitation of level of care, medical personnel involved, vital signs, laboratory data, consultation with a specialist if any (e.g. infectious diseases, internal medicine, anaesthesia), time on the ED, times to fluid administration and antibiotic treatment, other sepsis treatment such as corticosteroids, and highest serum lactate within 24 hours of admission. The highest priority given by the triage system RETTS (Rapid Emergency Triage and Treatment System) was documented [25, 26]. Sequential Organ Failure Assessment (SOFA) score [27] and National Early Warning Score (NEWS) 2 [28] were also calculated.

An expert group consisting of physicians specialised in infectious diseases judged the correctness of the antibiotic treatment based on local recommendations for empirical treatment of the suspected focus at that time, and the pathogen finally cultured. Doses of antibiotics, corrected for renal function, were also judged. Comorbidity scores according to the modified Charlson Comorbidity

For peer review only

Index, based on diagnoses obtained from patient medical records up to 24 months prior to the BSI, were later entered into the database.

Definitions

Blood culture: One set of blood cultures comprised one aerobic and one anaerobic blood culture bottle. It is recommended that at least two sets of blood cultures be taken simultaneously.

Positive blood culture: The isolations of microorganism/s (one or more bacterial or fungal isolates) from a set of blood cultures. Only bacterial or yeast isolates from initial blood cultures were considered; repeat isolates were excluded.

Non-significant pathogens: Microorganisms typically belonging to the skin microbiome (Coagulase-Negative Staphylococci, (CoNS), *Micrococcus* spp., *Bacillus* spp., *Corynebacterium* spp. and *Propionibacterium* spp.) were considered probable contaminants and excluded [29]. An exception was CoNS isolated from blood cultures taken from at least two different puncture sites on the same occasion, and with an identical resistance pattern.

Community-onset bloodstream infection (CO-BSI): A positive blood culture obtained within 48 hours after hospital admission; cultures from readmission within 3 days were excluded. In case of readmission within 4-30 days a different microorganism should have caused the new infection. Furthermore, patients who had received intravenous treatment or advanced nursing care at home such as parenteral nutrition, haemodialysis, or intravenous chemotherapy, within the previous 30 days were excluded.

For peer review only

Immunosupression: Patients were deemed immunocompromised if prescribed immunosuppressive medication or had a code number for an immunosuppressive disease according to the International Classification of Diseases, 10th edition (ICD-10).

Comorbidity: Comorbidity score was based on the updated Charlson Comorbidity Index [30-32] using ICD-10 diagnosis codes documented in the patient's medical record up to 24 months prior to date of admission (Supplementary Table 4).

Sepsis: The Sepsis-3 definition [33] was used *i.e.*, an increase by 2 points or more in the SOFA score due to infection.

Limitation of level of care: In some cases, the decision was made to limit the level of care. Such a decision influences treatment provided and thus mortality. This is an obvious confounding factor. Limitation of level of care set in the ED or within the first 24 h of admission was registered as: (a) withholding of intensive care (no ICU); (b) withholding of cardiopulmonary resuscitation (no CPR); and (c) withdrawal of all active treatment *i.e.*, end-of-life care (EoLC).

Microbiological methods

All microorganisms isolated were analysed at the species level. Microorganism identification and susceptibility testing were performed at the Clinical Microbiology Department, University Hospital, Linköping using matrix-assisted laser desorption ionisation time-of-flight mass spectrometry (MALDI-TOF MS). Antibiotic susceptibility classification used during study period was: Susceptible (S), Intermediate (I), and Resistant (R), according to the European Committee on Antimicrobial Susceptibility Testing (EUCAST) guidelines [34].

For peer review only

Ethics approval

The Regional Ethics Review Board in Linköping, Sweden, approved the study (Ref. no: 2010/160-31).

Patient and public involvement

There was no patient and/or public involvement in the design of the study.

Statistical analysis

Data are presented as mean and standard deviation (SD) or median and quartiles (Q1-Q3). We compared normally distributed quantitative variables using the Student *t* test and categorical variables using the chi^2 test or Fisher exact test as appropriate. Mann–Whitney U test and Kruskal Wallis test were used otherwise. A two-tailed p value < 0.05 was considered statistically significant. Correlations were analysed according to Pearson. Missing data were treated by pairwise deletion, and there was no imputation. Univariate and multivariable analyses were performed with logistic regression including the descriptive parameters that correlated to 30-day mortality. Two patients among the non-survivors had EoLC taken prior to admission to the hospital. This was considered to have had a major effect on early treatment and time to admission to hospital for these patients and they and their matched survivors were excluded from the analyses regarding time to admission and the multiple regression model. The statistics programme SPSS version 25 was used for the analyses.

For peer review only

Results

Demographic and clinical characteristics

A total of 2,356 BSI episodes were identified during the study period, of which 1,624 (69%) were CO-BSIs. The all-cause 30-day mortality rate of CO-BSI was 12.6% (n=205). Of these, 95% (n=195) met the inclusion criteria and were included in the study as non-survivors (case-group) and were matched with 195 survivors (control group) for gender, age, and microorganism. Almost 40% of the non-survivors died within 3 days after admission, and 80% within 7 days. Underlying comorbidity scores according to the updated Charlson Comorbidity Index were higher among non-survivors than survivors (3.2 vs 2.3, p=<0.01). Non-survivors were more often diagnosed with cancer (34% vs 23%, p=0.01) and metastatic carcinoma (14% vs 7%, p=0.02) compared to survivors. Limitation of level of care before admission was significantly more common among non-survivors, 14% vs 2% (Table 1 and Supplementary Table 1).

For peer review only

Table 1. Demographics, severity of diseases and antibiotic treatment

	Non-survivors n=195	Survivors n=195	p-value
**Demographics (%)**			
Male	106 (54)	106 (54)	>0.99
Mean age (SD)	78y (±13)	76y (±12)	0.20
Surgery within 30d	29 (15)	27 (14)	0.77
Immunosuppression	60 (31)	45 (23)	0.09
**Charlson (update weight) (SD)**	3.2 (2.99)	2.3 (2.36)	*0.01*
**Patients with any limitation of level of care before admission (%)**	28 (14)	4 (2)	*<0.01*
EoLC	2 (1)	0	0.20
No ICU	17 (9)	2 (1)	0.77
No CPR	27 (14)	4 (2)	0.09
**Ambulance transport (%)**	158 (81) n194	136 (70) n193	*0.01*
**Admission to hospital (%)**			
Emergency department	165 (85)	178 (91)	*0.04*
Specialist outpatient department	30 (15)	17 (9)	*0.04*
**Severity of disease (SD)**			
Habitual SOFA	0.8 (1.1) n185	0.4 (0.8) n193	*<0.01*
SOFA score on admission	4.2 (2.3) n161	2.3 (1.7) n160	*<0.01*
SOFA score at 24 h	6.8 (3.6) n 187	3.8 (2.7) n 195	*<0.01*
Maximum lactate level mmol/l first 24 hr	4.6 (4.0) n 122	2.9 (1.9) n 110	*<0.01*
NEWS 2	7.1 (4.0) n 163	5.0 (3.4) n 181	*<0.01*
**Sepsis (%)**			
Sepsis on admission	119 (75) n158	82 (52) n158	*<0.01*
Sepsis at 24 h	176 (95) n185	152 (79) n193	*<0.01*
**ICU care within 24 hours (%)**	22 (11.3)	9 (4.6)	*0.01*
**Antibiotic treatment**			
Antibiotic administration (%)	186 (96) n193	192 (99.5) n193	0.037
Time (hours) to antibiotics from admission median, (Q1-Q3)	2.8 (1.4-5.3) n185	3.0 (1.4-6.2) n192	0.58
First dose antibiotic within one hour (%)	34 (18) n185	30 (16) n192	0.48
Appropriate empirical antibiotics (%)	162 (87) n186	185 (96) n192	*<0.01*
Appropriate antibiotics based on cultured bacteria (%)	155 (83) n186	168 (88) n192	0.25
**Intravenous fluids in the ED (%)**	124 (76) n163	116 (68) n171	0.09

Data are presented as no. (%) or mean (SD). Pearson chi2, Fisher's exact test or T-test, as appropriate. P values < 0.05 are shown in italics. Time indications are calculated with median, interquartile 25th to 75th percentile range (Q1-Q3) and Mann–Whitney. Two non-survivors with End-of-life care (EoLC) on arrival at hospital, and their controls were excluded from the analysis regarding antibiotic treatment and intravenous fluids.

For peer review only

Microbiology

There were 407 isolates from 390 cases. *Staphylococcus aureus* was the most frequently isolated primary pathogen (27%), followed by *Escherichia coli* (21%), *Streptococcus pneumoniae* (8%) and *Klebsiella pneumoniae* (7%). *Candida* spp. were observed in 2% of cases. Polymicrobial CO-BSI was found in 9 non-survivors and 8 survivors. Microbiological findings are summarised in Supplementary Table 2.

Prehospital data

Of the 390 patients, 61% (115 non-survivors and 121 survivors) had taken contact with a prehospital healthcare facility for infection-related symptoms within 7 days prior to admission to hospital. Forty-five per cent of the patients had ≥2 prehospital contacts. Forms of prehospital contact were phone contact with a PHCC (47%), phone contact with NHG (31%), and visit to a PHCC (21%). Fever was the primary reason for prehospital contact (37%). Rapid deterioration in general condition was the second most common reason (32%) and was more common among non-survivors than survivors (41% vs 24%, $p<0.02$) (Table 2). Phone contact with a PHCC was the more common form of first contact among non-survivors (41% non-survivors vs 17% survivors, $p=0.01$), whereas phone contact with NHG was more common among survivors (7% non-survivors vs 31% survivors, $p=<0.01$) (Table 2).

For peer review only

Table 2. Characteristics of the prehospital phase

	Non-survivors n=195	Survivors n=195	p-value
**First prehospital contact site (%)**			
Phone to NHG	14 (7)	60 (31)	*<0.01*
Phone to a PHCC	79 (41)	33 (17)	*<0.01*
Patient visit to a PHCC	22 (11)	28 (14)	0.36
No prehospital contact	80 (41)	74 (38)	0.534
**Multiple prehospital contacts (≥2) (%)**	64 (33)	41 (21)	*0.01*
**Reason for first prehospital contact (number) (%)**	n115	n121	
Fever	44 (38)	43 (36)	>0.665
Chills	13 (11)	13 (11)	>0.89
"Found on the floor"	4 (3)	0	*0.04*
Gastrointestinal symptoms	16 (14)	23 (19)	0.29
Cough	3 (3)	13 (11)	*0.01*
Dyspnea/breathing difficulties	23 (20)	15 (12)	0.11
Urinary tract symptoms	14 (12)	22 (18)	0.20
Rapid deterioration in general condition	47 (41)	29 (24)	*<0.01*
Fatigue	3 (3)	15 (12)	*<0.01*
**No prehospital contact (%)**	80 (42)	74 (38)	0.534
**Time from first prehospital contact to admission h, median (Q1-Q3)**	13 (5.9-51.6) n113	7.2 (3.3-24) n119	*<0.01*
Phone to NHG	4 (1.0-8.7) n13	3.6 (1.3-7.3) n58	0.64
Phone to a PHCC	24 (6.8-72) n78	12.5 (5.9-24) n33	0.06
Visit to a PHCC	11 (6.6-77) n22	12.5 (6.4-66) n28	0.75
**Time from first prehospital contact to admission, intervals (%)**	n193	n193	
0-6 hours	29 (15)	49 (25)	*0.011*
6-12 hours	26 (14)	30 (16)	0.563
12-24 hours	12 (6)	21 (11)	0.101
>24 hours	46 (24)	19 (10)	*<0.001*
No prehospital contact	80 (41)	74 (38)	0.534

Data are presented as no. (%) or mean (SD). Pearson chi2, Fisher's exact test or T-test, as appropriate. P values < 0.05 are shown in italics. Time indications are calculated with median, interquartile 25th to 75th percentile range (Q1-Q3) and Mann–Whitney U. Reason for first prehospital contact: more than one reason possible. NHG = national healthcare guide. PHCC=primary healthcare centre

Time from first infection-related prehospital contact till hospital admission

In total, the median time from first prehospital contact till hospital admission was 8 hours (4-27). This was significantly longer for non-survivors 13 hours (6-52) vs 7.2 hours (3-24) for survivors, p=0.01 (Table 2). For the study group as a whole, there were significant differences in time to

For peer review only

For peer review only - http://bmjopen.bmj.com/site/about/guidelines.xhtml

admission depending on form of first contact, with shorter median time to admission after telephone contact with the NHG 3.8 hours (1.3-7.7) compared to phone contact with a PHCC 24.0 hours (6.8-48) or visit to a PHCC 11.3 hours (6.7-72) ($p<0.01$).

Hospital data

A total of 88% were admitted to hospital via the ED (85% non-survivors vs 91% survivors, p=0.04), other patients were admitted via a specialist outpatient department. Hospital admission after ambulance transport was more common among non-survivors (81% vs 70%, p=0.01) (Table 1). The most common sign reported when admitted to hospital was fever (54%) (Supplementary Table 5). The most common infection focus was a urinary tract infection (24%) followed by infection with unknown focus (23%), and respiratory tract infection (22%). There were no differences between non-survivors and survivors regarding primary focus of infection (Supplementary Table 3).

On the ED, 159 non-survivors and 161 survivors were triaged with a life-threatening or potentially life-threatening condition according to the RETTS score (RETTS and NEWS 2 scores are provided in Supplementary Table 5). Abnormalities in respiratory function, haemodynamics, and neurological function were significantly more prominent among non-survivors. SOFA score on admission and at 24 hours was higher among non-survivors 4.2 (SD: 2.3) vs 2.3 (SD: 1.7), ($p<0.01$) and 6.8 (3.6) vs 3.8 (2.7) ($p<0.01$) respectively) (Table 1). Vital signs and most laboratory test results indicated a more serious condition in non-survivors compared to survivors. (Supplementary Table 3). On admission to hospital, 75% of non-survivors and 52% of survivors ($p<0.01$) fulfilled the Sepsis 3 criteria, and within 24 hours the corresponding figures were 95% of non-survivors and 79% of survivors ($p<0.01$). In all, 11% of non-survivors received ICU care within 24 hours compared to 5% of survivors (p=0.01) (Table 1).

For peer review only

Antibiotic treatment was initiated in 96% of non-survivors and 99.5% of survivors, (p=0.04). The median time to start did not differ between the groups, non-survivors 2.8 hours (1.4-5.3) and survivors 3.0 hours (1.4-6.2). Antibiotic administration was started within one hour in 18% of non-survivors and 16% of survivors (p=0.48). More survivors received appropriate empiric antimicrobial therapy than non-survivors (87% non-survivors and 96% survivors, p<0.01). Appropriate choice of antibiotics based on subsequent blood culture did not differ significantly between the groups (82% non-survivors vs 88% survivors) (Table 1).

Analysis of risk factors for 30-day mortality in CO-BSI

In the univariate logistic regression several risk factors associated with 30-day mortality were observed: comorbidity score (updated Charlson Index) OR = 1.13 (CI 1.05-1.22), p<0.01, prehospital delay OR = 1.26 (CI 1.07-1.47), p=0.01, severity of illness (SOFA score on admission) OR = 1.60 (CI 1.40-1.83), p<0.01 and inadequate empirical antibiotic treatment OR = 3.92 (CI 1.64-9.33), p<0.01 (Table 3).

For peer review only

Table 3. Risk factors for 30-day mortality

	Univariate analysis			Multivariable analysis		
**Risk factor**	**OR**	**95% CI**	**p-value**	**OR***	**95% CI**	**p-value**
**Time from first prehospital contact to admission (intervals)**	1.26	1.07-1.47	*0.01*			
0-6h	1**			1**		
6-12h	1.46	0.73-2.94	0.28	1.35	0.48-3.79	0.56
12-24h	0.97	0.42-2.25	0.94	0.56	0.15-2.10	0.39
>24h	4.09	2.02-8.28	*<0.01*	6.17	2.19-17.38	*<0.01*
No prehospital contact	1.83	1.05-3.19	*0.03*	2.56	1.02-6.41	0.045
**Updated Charlson**	1.13	1.05-1.22	*<0.01*	1.02	0.91-1.15	0.70
**Ambulance transport**	1.84	1.14-2.97	*0.01*	1.59	0.68-3.71	0.29
**Admission through ED**	0.55	0.29-1.03	0.06	1.35	0.43-4.25	0.61
**In SOFA score**	1.60	1.40-1.83	*<0.01*	1.35	1.16-1.57	*<0.01*
**Inadequate empirical antibiotics**	3.92	1.64-9.33	*<0.01*	5.50	1.62-18.63	*0.01*
**Rapid deterioration in general condition**	1.79	1.06-3.02	*0.03*	2.27	0.98-5.28	0.06
**Any care restrictions before or within 24h after admission**	9.95	5.95-16.64	*<0.01*	10.37	4.96-21.67	*<0.01*
**Matching**	1	0.99-1.00	>0.99	1	0.99-1.0	0.20

* Multivariable binomial regression analysis, ** Reference.

In the multivariable model of 30-day mortality, prehospital delay >24 hours was significantly related to mortality OR = 6.17 (CI 2.19-17.38), p<0.01. This was followed by inadequate empirical antibiotic treatment OR = 5.50 (CI 1.62-18.63), p=0.01, no prehospital contact OR = 2.56 (CI 1.02-6.41), p=0.045, and severity of illness (SOFA score on admission) OR = 1.35 (CI 1.16-1.57), p<0.01 (Table 3).

Discussion

This study revealed that prehospital delay had a major impact on mortality among patients with CO-BSI. Non-survivors were more seriously ill when admitted to hospital, with significantly higher SOFA scores and more signs of sepsis. Although initial care at the hospital and time to antibiotic

For peer review only

treatment were comparable between non-survivors and survivors (for some variables even better among non-survivors) this was not enough to compensate for the negative effect of prehospital delay.

CO-BSI patients form a heterogeneous group in which underlying disease, gender, age, and appropriate management vary significantly. The severity of bloodstream infection ranges from asymptomatic bacteraemia to fulminant sepsis or septic shock. To our knowledge, this is the first study to focus on a well-defined population of adult patients with CO-BSI focusing on prehospital care prior to hospital admission. In previous study by Holmbom *et al*, gender, age, and pathogen were risk factors for mortality in BSI [7, 11], so these were used when matching patients to enable evaluation of other potential risk factors in this study. Compared to previous studies and reports on sepsis and CO-BSI, the distribution of pathogens in the present study was different [7, 35, 36], with *S.aureus* being the most common. This was probably a result of the study design *i.e.,* inclusion of patients dying within 30 days and matched controls, since *S.aureus* BSI has such a high mortality rate [37, 38].

Delay in antibiotic treatment has previously been shown to have a clear association with increased mortality in progressive sepsis and septic shock [13, 15, 17, 35, 39, 40]. However, delay prior to ambulance transport or coming to the ED, and causes of delay have hardly been investigated [24]. Strategies for early identification of sepsis in prehospital care, and measures to increase survival from sepsis have received much attention, and several strategies and screening tools have been investigated with varied results [18, 21, 41]. Among the patients in the present study who took prehospital contact with the healthcare service, form of first prehospital contact affected prehospital delay. Telephone contact with the NHG was associated with shorter time to hospital admission than phone contact or visit to the local PHCC. The reason for this remains unclear. Possible factors include: 1. Compliance to the NHG computer-based decision algorithm designed for acute illness, as well as nurses more experienced in giving advice over the phone; 2. More liberal attitude of

For peer review only

NHG nurses to send patients to the ED; 3. Non-survivors have higher comorbidity scores and tend to seek initial contact with their PHCC. Frail patients have also greater difficulty in describing their symptoms, making it more difficult for the primary healthcare provider to evaluate the seriousness of their condition; 4. Whereas the NHG gives advice directly, it is possible for the PHCC to delay clinical assessment until later the same or next day. In fact, the call-you-back system used by PHCCs may have caused even more delay than seen in this study since we did not have access to when the first phone call from the patient was made. Instead, we used the time the phone-call back to the patient was made. In conclusion, the BSI healthcare process and its outcome varies according to the form of healthcare provider the patient first contacts.

We also looked for factors facilitating earlier identification of patients with increased risk for death. The comorbidity score (updated Charlson Comorbidity Index) was higher among non-survivors, with higher rates of cancer and metastatic carcinoma. These conditions are associated not only with increased mortality risk in general but also with BSI itself [7, 11, 42]. In prehospital records, “found on the floor” and “rapid deterioration in general condition” were more often reported among non-survivors. Since both reflect a seriously ill person, the patient is by proxy identified as a patient at risk. Although early prehospital identification of patients at high risk for sepsis or mortality is complex [41], we confirm the findings of previous studies [7, 11, 42] that taking signs and symptoms of infection in cancer patients seriously is crucial. Otherwise, we found no patient-specific factors that could be of help in identifying patients with increased mortality risk.

Many studies on sepsis, especially prior to Sepsis 3, have had patients with a wide spectrum of disease severity. This has certainly contributed to differences in results regarding the impact of time to antibiotic treatment on outcome [13-15, 17]. Our study comprised patients with CO-BSI, not true sepsis, and thus even more likely to be heterogeneous. Even so, 75% of non-survivors and 52% of

For peer review only

survivors had sepsis on admission to the ED. The severity of illness on admission to hospital was greater in non-survivors and may be explained by prehospital delay.

Previous studies have shown the importance of rapid detection and initiation of adequate antibiotic treatment in sepsis and especially septic shock [13-15, 17, 35]. Nevertheless, using the RETTS triage system [25, 33], only half of the non-survivors were captured by the highest priority (red). Furthermore, priorities red plus orange together would still have failed to detect one third. Likewise, when using NEWS 2 with the proposed cut-off score of 5 [28, 43], at least one third of the non-survivors would have remained undetected as patients at risk for sepsis in the initial triage. Triage is useful when escalating the care of patients already identified with sepsis, as shown by Rosenqvist *et al* [22], but we lack a valid, simple, highly predictive scoring system to detect sepsis [44, 45]. Surviving Sepsis Guidelines 2016 recommend that antibiotics be administered within one hour of diagnosing sepsis [46]. This was not achieved in our material where only 16% of non-survivors and 18% of survivors received the first dose of antibiotics within one hour. Appropriate empirical antibiotic treatment according to local guidelines was more common among survivors ($p<0.01$), while appropriate antibiotic according to subsequent blood cultures was not. This illustrates that not only the administration of correct empirical antibiotics according to recommendations but also a holistic view of the patient, including suspected focus and need of source control, are both important parts of initial management. However, this study was not designed to evaluate the correctness of antimicrobial therapy. For those receiving antibiotics, there was no significant difference in time to administration and no difference in adequacy of antibiotic treatment related to microorganisms cultured. More rapid initiation of antibiotic treatment in hospital among non-survivors may have improved their outcome but this did not seem to be the main explanation for mortality in our study. However, prehospital delay causes delay to antibiotic administration and probably worsens the outcome.

For peer review only

Limitations

To our knowledge, this is the first study on a well-defined population of adult patients with CO-BSI, focusing on prehospital care prior to hospital admission, but it has limitations.

1. Though non-survivors and survivors were matched for gender, age (+/- 10 years), and microorganism, microorganism resistance patterns were not considered when matching non-survivors and survivors. However, since the prevalence of resistant bacteria in Sweden is low, this could only have had a minor influence on the results. Furthermore, when analysing correct antimicrobial therapy based on microorganisms cultured and their resistance patterns, there was no significant difference between the groups.
2. We only analysed the correctness of empirical antibiotic treatment, while early changes in therapy were not considered.
3. Other causes of death such as myocardial infarction, respiratory failure or pulmonary embolism as primary cause of death were possible, but such conditions would likely be related to the underlying infection, and since 95% of the cases had a fulminant sepsis within 24 hours this would be the major predisposing factor.
4. The only differences in prehospital management affecting outcome in this study were the timing of events. Other variables require further research.

Conclusion

In this case-control study, prehospital delay was found to be an important risk factor for 30-day all-cause mortality associated with CO-BSI. Effective guidelines to aid recognition of patients likely to develop sepsis in prehospital and early hospital care, and an effective prehospital medical advice service, could help to make significant progress in the care of patients with bloodstream infection.

For peer review only

Acknowledgements

Thanks to senior anaesthetist Peter Cox for proof reading.

Competing interests

None declared.

Author Contributions

MH, HH and ÅÖB conceived and designed the study. MH, MA, DE, ÅÖB and MF analysed the data. MH and MA wrote the manuscript. All authors contributed to the discussion and reviewed the manuscript. All authors commented and approved the final version of the paper.

Data Availability

All relevant data are within the manuscript and its Supplementary files.

Funding

This work was supported by Östergötland Count Council (Award/grant number: N/A).

References

1. Damiani, E., et al., *Effect of performance improvement programs on compliance with sepsis bundles and mortality: a systematic review and meta-analysis of observational studies.* PLoS One, 2015. **10**(5): p. e0125827.
2. Dellinger, R.P., et al., *Surviving sepsis campaign: international guidelines for management of severe sepsis and septic shock: 2012.* Crit Care Med, 2013. **41**(2): p. 580-637.
3. Gupta, R.G., et al., *Early goal-directed resuscitation of patients with septic shock: current evidence and future directions.* Crit Care, 2015. **19**: p. 286.
4. Rivers, E., et al., *Early goal-directed therapy in the treatment of severe sepsis and septic shock.* N Engl J Med, 2001. **345**(19): p. 1368-77.
5. Wisplinghoff, H., et al., *Nosocomial bloodstream infections in US hospitals: analysis of 24,179 cases from a prospective nationwide surveillance study.* Clin Infect Dis, 2004. **39**(3): p. 309-17.
6. Goto, M. and M.N. Al-Hasan, *Overall burden of bloodstream infection and nosocomial bloodstream infection in North America and Europe.* Clin Microbiol Infect, 2013. **19**(6): p. 501-9.

For peer review only

For peer review only - http://bmjopen.bmj.com/site/about/guidelines.xhtml

7. Holmbom, M., et al., *14-Year Survey in a Swedish County Reveals a Pronounced Increase in Bloodstream Infections (BSI). Comorbidity - An Independent Risk Factor for Both BSI and Mortality.* PLoS One, 2016. **11**(11): p. e0166527.
8. Mehl, A., et al., *Burden of bloodstream infection in an area of Mid-Norway 2002-2013: a prospective population-based observational study.* BMC Infect Dis, 2017. **17**(1): p. 205.
9. Skogberg, K., et al., *Population-based burden of bloodstream infections in Finland.* Clin Microbiol Infect, 2012. **18**(6): p. E170-6.
10. Sogaard, M., et al., *Temporal changes in the incidence and 30-day mortality associated with bacteremia in hospitalized patients from 1992 through 2006: a population-based cohort study.* Clin Infect Dis, 2011. **52**(1): p. 61-9.
11. Holmbom, M., et al., *Low incidence of antibiotic-resistant bacteria in south-east Sweden: An epidemiologic study on 9268 cases of bloodstream infection.* PLoS One, 2020. **15**(3): p. e0230501.
12. Laupland, K.B. and D.L. Church, *Population-based epidemiology and microbiology of community-onset bloodstream infections.* Clin Microbiol Rev, 2014. **27**(4): p. 647-64.
13. Ferrer, R., et al., *Empiric antibiotic treatment reduces mortality in severe sepsis and septic shock from the first hour: results from a guideline-based performance improvement program.* Crit Care Med, 2014. **42**(8): p. 1749-55.
14. Kumar, A., et al., *Initiation of inappropriate antimicrobial therapy results in a fivefold reduction of survival in human septic shock.* Chest, 2009. **136**(5): p. 1237-1248.
15. Liu, V.X., et al., *The Timing of Early Antibiotics and Hospital Mortality in Sepsis.* Am J Respir Crit Care Med, 2017. **196**(7): p. 856-863.
16. Vucelic, V., et al., *Sepsis and septic shock - an observational study of the incidence, management, and mortality predictors in a medical intensive care unit.* Croat Med J, 2020. **61**(5): p. 429-439.
17. Kumar, A., et al., *Duration of hypotension before initiation of effective antimicrobial therapy is the critical determinant of survival in human septic shock.* Crit Care Med, 2006. **34**(6): p. 1589-96.
18. Lane, D.J., et al., *Screening strategies to identify sepsis in the prehospital setting: a validation study.* CMAJ, 2020. **192**(10): p. E230-E239.
19. Park, J., et al., *Emergency medical service personnel need to improve knowledge and attitude regarding prehospital sepsis care.* Clin Exp Emerg Med, 2017. **4**(1): p. 48-55.
20. Sjosten, O., et al., *The prehospital assessment of patients with a final hospital diagnosis of sepsis: Results of an observational study.* Australas Emerg Care, 2019. **22**(3): p. 187-192.
21. Smyth, M.A., S.J. Brace-McDonnell, and G.D. Perkins, *Identification of adults with sepsis in the prehospital environment: a systematic review.* BMJ Open, 2016. **6**(8): p. e011218.
22. Rosenqvist, M., et al., *Improved Outcomes After Regional Implementation of Sepsis Alert: A Novel Triage Model.* Crit Care Med, 2020. **48**(4): p. 484-490.
23. Latten, G.H.P., et al., *Characteristics of the prehospital phase of adult emergency department patients with an infection: A prospective pilot study.* PLoS One, 2019. **14**(2): p. e0212181.
24. Seymour, C.W., et al., *Delays From First Medical Contact to Antibiotic Administration for Sepsis.* Crit Care Med, 2017. **45**(5): p. 759-765.
25. Widgren, B.R. and M. Jourak, *Medical Emergency Triage and Treatment System (METTS): a new protocol in primary triage and secondary priority decision in emergency medicine.* J Emerg Med, 2011. **40**(6): p. 623-8.
26. Widgren, B.R., M. Jourak, and A. Martinius, *[New accurate triage method. METTS-A yields basis for priority level decisions].* Lakartidningen, 2008. **105**(4): p. 201-4.
27. Vincent, J.L., et al., *The SOFA (Sepsis-related Organ Failure Assessment) score to describe organ dysfunction/failure. On behalf of the Working Group on Sepsis-Related Problems of the European Society of Intensive Care Medicine.* Intensive Care Med, 1996. **22**(7): p. 707-10.

For peer review only

28. *Royal College of Physicians. National Early Warning Score (NEWS) 2: Standardising the assessment of acute-illness severity in the NHS. Updated report of a working party. London: RCP, 2017.*
29. Ammerlaan, H.S., et al., *Secular trends in nosocomial bloodstream infections: antibiotic-resistant bacteria increase the total burden of infection.* Clin Infect Dis, 2013. **56**(6): p. 798-805.
30. Quan, H., et al., *Updating and validating the Charlson comorbidity index and score for risk adjustment in hospital discharge abstracts using data from 6 countries.* Am J Epidemiol, 2011. **173**(6): p. 676-82.
31. Quan, H., et al., *Coding algorithms for defining comorbidities in ICD-9-CM and ICD-10 administrative data.* Med Care, 2005. **43**(11): p. 1130-9.
32. Sundararajan, V., et al., *New ICD-10 version of the Charlson comorbidity index predicted in-hospital mortality.* J Clin Epidemiol, 2004. **57**(12): p. 1288-94.
33. Singer, M., et al., *The Third International Consensus Definitions for Sepsis and Septic Shock (Sepsis-3).* JAMA, 2016. **315**(8): p. 801-10.
34. *The European Committee on Antimicrobial Susceptibility Testing. Breakpoint tables for interpretation of MICs and zone diameters. Version 8.1, 2018.* http://www.eucast.org.
35. Andersson, M., et al., *Delay of appropriate antibiotic treatment is associated with high mortality in patients with community-onset sepsis in a Swedish setting.* Eur J Clin Microbiol Infect Dis, 2019.
36. Henriksen, D.P., et al., *Incidence rate of community-acquired sepsis among hospitalized acute medical patients-a population-based survey.* Crit Care Med, 2015. **43**(1): p. 13-21.
37. Jacobsson, G., E. Gustafsson, and R. Andersson, *Outcome for invasive Staphylococcus aureus infections.* Eur J Clin Microbiol Infect Dis, 2008. **27**(9): p. 839-48.
38. Melzer, M. and C. Welch, *Thirty-day mortality in UK patients with community-onset and hospital-acquired meticillin-susceptible Staphylococcus aureus bacteraemia.* J Hosp Infect, 2013. **84**(2): p. 143-50.
39. Whiles, B.B., A.S. Deis, and S.Q. Simpson, *Increased Time to Initial Antimicrobial Administration Is Associated With Progression to Septic Shock in Severe Sepsis Patients.* Crit Care Med, 2017. **45**(4): p. 623-629.
40. Seymour, C.W., et al., *Time to Treatment and Mortality during Mandated Emergency Care for Sepsis.* N Engl J Med, 2017. **376**(23): p. 2235-2244.
41. Lane, D., et al., *Prehospital management and identification of sepsis by emergency medical services: a systematic review.* Emerg Med J, 2016. **33**(6): p. 408-13.
42. Velasco, E., et al., *Bloodstream infection surveillance in a cancer centre: a prospective look at clinical microbiology aspects.* Clin Microbiol Infect, 2004. **10**(6): p. 542-9.
43. Corfield, A.R., et al., *Utility of a single early warning score in patients with sepsis in the emergency department.* Emerg Med J, 2014. **31**(6): p. 482-7.
44. Churpek, M.M., et al., *Quick Sepsis-related Organ Failure Assessment, Systemic Inflammatory Response Syndrome, and Early Warning Scores for Detecting Clinical Deterioration in Infected Patients outside the Intensive Care Unit.* Am J Respir Crit Care Med, 2017. **195**(7): p. 906-911.
45. Gerry, S., et al., *Early warning scores for detecting deterioration in adult hospital patients: systematic review and critical appraisal of methodology.* BMJ, 2020. **369**: p. m1501.
46. Rhodes, A., et al., *Surviving Sepsis Campaign: International Guidelines for Management of Sepsis and Septic Shock: 2016.* Intensive Care Med, 2017. **43**(3): p. 304-377.

For peer review only

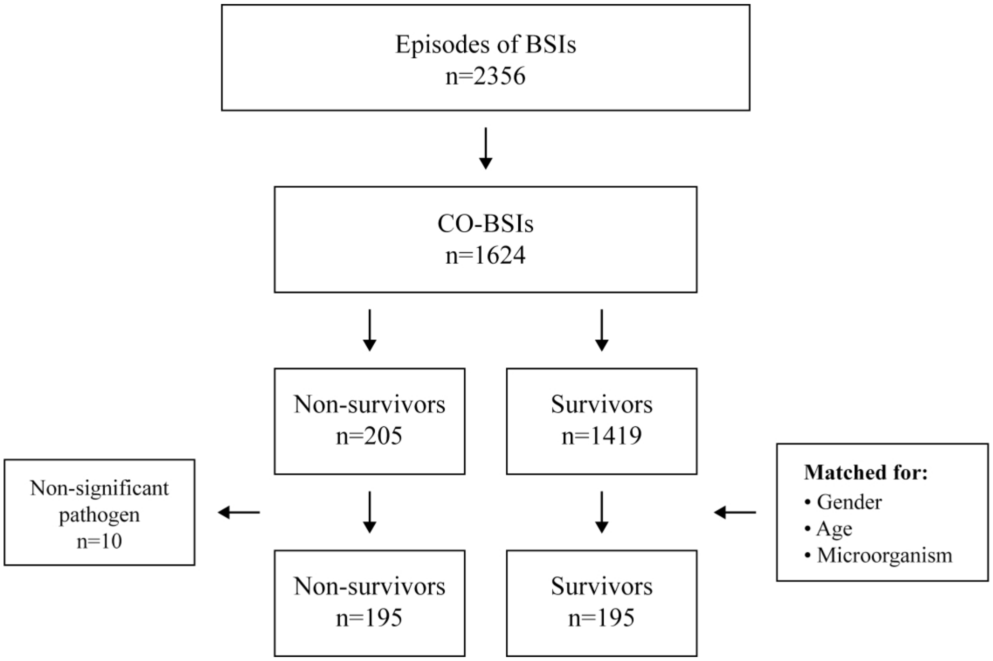

Figure 1. Flow chart of the study

Supplementary Table 1. Distribution of comorbidities

	**Non-survivors n=195**	**Survivors n=195**	**p-value**
**Comorbidity (%)**			
Myocardial infarction	31 (16)	41 (21)	0.19
Congestive heart failure	56 (29)	50 (26)	0.50
Peripheral vascular disease	14 (7)	16 (8)	0.70
Cerebrovascular disease	30 (15)	28 (14)	0.78
Dementia	15 (8)	11 (6)	0.42
Chronic pulmonary disease	35 (18)	33 (17)	0.79
Connective Tissue Disease-Rheumatic Disease	13(7)	22(11)	0.11
Peptic ulcer disease	3 (2)	3(2)	>0.99
Mild liver disease	8 (4)	9 (5)	0.80
Diabetes without chronic complications	43(22)	62(32)	*0.03*
Diabetes with chronic complications	8 (4)	9 (5)	0.80
Paraplegia and hemiplegia	13 (7)	11 (6)	0.67
Renal disease	34 (17)	25 (13)	0.20
Cancer	67 (34)	44 (23)	*0.01*
Moderate or severe liver disease	9 (5)	3 (2)	0.08
Metastatic carcinoma	28 (14)	14 (7)	*0.02*
HIV/AIDS	0	0	-
Charlson (update weight) (SD)	3.2 (2.99)	2.3 (2.36)	*0.01*

Data are presented as no. (%) or mean (SD). Pearson chi2, Fisher's exact test or T-test, as appropriate.

For peer review only

Supplementary Table 2. Distribution of primary pathogen*

**Gram negative**	**Non-survivors n=195**	**Survivors n=195**	**%**
**Enterobacterales**			
Escherichia coli	41	41	21
Klebsiella pneumoniae	14	14	7
Klebsiella oxytoca	3	3	2
Enterobacter cloacae	2	2	1
Proteus Mirabilis	4	4	2
Haemophilus influenzae	4	4	2
**Other than Enterobacterales**			
Pseudomonas aeruginosa	7	7	4
**Others**	7	7	4
**Gram positives**			
**Enterococci**			
Enterococcus faecalis	6	6	3
Enterococcus faecium	5	5	3
**Staphylococci**			
Staphylococcus aureus	52	52	27
Staphylococcus spp	5	5	3
**Streptococci**			
Streptococcus pneumoniae	16	16	8
Streptococcus pyogenes (A)	1	1	1
Streptococcus agalactiae (B)	4	4	2
Streptococcus group C,G	5	5	3
Streptococcus spp	11	11	6
***Candida* spp**	4	4	2
**Others**	4	4	2
**Polymicrobial-BSI**	9	8	4

*Primary pathogen reported in the table. In Polymicrobial-BSI, an expert group (physicians specialised in infectious diseases) has evaluated which pathogen is primary.

For peer review only

Supplementary Table 3. Hospital data

	Non-survivors n=195	Survivors n=195	p-value
**Vital signs (SD)***			
Body temp (BT)	37.6 (1.3) n189	38.2 (1.1) n194	*<0.01*
Systolic blood pressure (SBP)	112 (28) n190	131 (28) n193	*<0.01*
Diastolic blood pressure (DBP)	65 (18) n188	72 (17) n192	*<0.01*
Pulse rate (HR)	102 (26) n190	97 (22) n192	*0.04*
Respiratory rate (RR)	27 (10) n184	23 (7) n189	*<0.01*
Blood O_2 saturation (SPO_2)	92 (9) n187	95 (4) n192	*<0.01*
PaO_2/FiO_2	42 (21) n183	51 (20) n187	*<0.01*
Reaction level scale (RLS)	1.4 (0.9) n187	1.1 (0.46) n192	*<0.01*
**Laboratory median (Q1-Q3)**			
CRP	180 (87-304) n195	138 (50-225) n192	*<0.01*
LEU	13 (8-19) n195	13(10-17) n191	0.42
TPK	194 (130-292) n195	212 (156-280) n191	0.09
CREA	143 (94-216) n191	98 (74-139) n190	*<0.01*
eGFR MDRD	36 (22-58) n186	58 (35-74) n186	*<0.01*
Albumin	23 (20-28) n109	29 (25-32) n100	*<0.01*
B-Erytrocyter	4 (3-4) n195	4 (4-5) n191	*0.04*
B-MCV	95 (91-101) n195	93 (90-97) n191	*<0.01*
Hb	116 (101-136) n195	121 (109-138) n191	*0.02*
Procalcitonin	10 (2-54) n34	4 (1-31) n26	0.325
**Primary focus of infection (%)**			
Respiratory tract	39 (20)	46 (24)	0.39
Urinary tract	42 (22)	50 (26)	0.34
Skin and soft tissue	19 (10)	20 (10)	0.86
Abdomen	12 (6)	18 (9)	0.25
CNS	-	2 (1)	0.50*
Indwelling catheter	1 (1)	0	>0.99*
Endocarditis	1 (1)	1 (1)	>0.99
Unknown	46 (24)	43 (22)	0.72
Missing data	35 (18)	15 (8)	

Data are presented as no. (%) or mean (SD). Pearson chi2, Fisher's exact test or T-test, as appropriate. Laboratory data are calculated with median, interquartile 25th to 75th percentile range (Q1-Q3) and Mann–Whitney U. * Vital signs first registered at admission.

For peer review only

Supplementary Table 4. Charlson Comorbidity Index

Comorbid Condition	ICD-9-CM Diagnosis Codes	ICD-10-CA Diagnosis Codes	Weight	Updated Weight
**Myocardial Infarction**	410, 412	I21, I22, I25.2	1	0
**Congestive Heart Failure**	398.91, 402.01, 402.11, 402.91, 404.01, 404.03, 404.11, 404.13, 404.91, 404.93, 425.4–425.9, 428 (hosp), 398, 402, 425, 428 (med)	I09.9, I11.0, I13.0, I13.2, I25.5, I42.0, I42.5–I42.9,I43, I50, P29.0	1	2
**Peripheral Vascular disease**	093.0, 437.3, 440, 441, 443.1–443.9, 447.1, 557.1, 557.9, V43.3 (hosp) 440, 441, 443, 447, 557 (med)	I70, I71, I73.1, I73.8, I73.9, I77.1, I79.0, I79.2, K55.1, K55.8, K55.9, Z95.8, Z95.9	1	0
**Cerebrovascular Disease**	362.34, 430–438 (hosp) 430–438 (med)	G45, G46, H34.0, I60–I69	1	0
**Dementia**	290, 294.1, 331.2 (hosp) 290, 294, 331 (med)	F00–F03, F05.1, G30, G31.1	1	2
**Chronic Pulmonary Disease**	416.8, 416.9, 490–505, 506.4, 508.1, 508.8 (hosp), 416, 490–496, 500–505 (med)	I27.8, I27.9,J40–J47, J60–J67 J68.4, J70.1, J70.3	1	1
**Connective Tissue Disease-Rheumatic Disease**	446.5, 710.0–710.4, 714.0–714.2, 714.8, 725 (hosp), 446, 710, 714, 725 (med)	M05, M06, M31.5, M32–M34, M35.1, M35.3, M36.0	1	1
**Peptic Ulcer**	531–534	K25–K28	1	0
**Mild Liver Disease**	070.22, 070.23, 070.32, 070.33, 070.44, 070.54, 070.6, 070.9, 570, 571, 573.3, 573.4, 573.8, 573.9, V42.7 (hosp) 070, 570, 571, 573 (med)	B18, K70.0–K70.3, K70.9, K71.3–K71.5, K71.7, K73, K74, K76.0, K76.2–K76.4, K76.8, K76.9, Z94.4	1	2
**Diabetes without Chronic Complications**	250.0–250.3, 250.8, 250.9 (hosp) 250 (med)	E10.0, E10.1, E10.6, E10.8, E10.9, E11.0, E11.1, E11.6, E11.8, E11.9, E12.0, E12.1, E12.6, E12.8, E12.9, E13.0, E13.1, E13.6, E13.8, E13.9, E14.0, E14.1, E14.6, E14.8, E14.9	1	0
**Diabetes with Chronic Complications**	250.4–250.7 (med n/a)	E10.2–E10.5, E10.7, E11.2–E11.5, E11.7, E12.2–E12.5, E12.7, E13.2–E13.5, E13.7, E14.2–E14.5, E14.7	2	1

For peer review only

**Paraplegia or Hemiplegia**	334.1, 342, 343, 344.0–344.6, 344.9 (hosp), 334, 342–344 (med)	G04.1, G11.4, G80.1, G80.2, G81, G82, G83.0–G83.4, G83.9	2	2
**Renal Disease**	403.01, 403.11, 403.91, 404.02, 404.03, 404.12, 404.13, 404.92, 404.93, 582, 583.0–583.7, 585, 586, 588.0. V42.0, V45.1, V56 (hosp), 403, 582, 583, 585, 586, 588, V56 (med)	I12.0, I13.1, N03.2–N03.7, N052–N05.7, N18, N19, N25.0, Z49.0–Z49.2, Z94.0, Z99.2	2	1
**Cancer ***	140–172, 174–195.8, 200–208, 238.6 (hosp), 140–172, 174–195, 200–208, 238 (med)	C00–C26, C30–C34, C37–C41, C43, C45–C58, C60–C76, C81–C85, C88, C90–C97	2	2
**Moderate or Severe Liver Disease**	456.0–456.2, 572.2–572.4, 572.8 (hosp), 456, 572 (med)	I85.0, I85.9, I86.4, I98.2, K70.4, K71.1, K72.1, K72.9, K76.5–K76.7,	3	4
**Metastatic Carcinoma****	196–199	C77–C80	6	6
**HIV/AIDS**	042–044	B20–B22, B24	6	4

Abbreviations: AIDS, acquired immunodeficiency syndrome; HIV, human immunodeficiency virus. * Including lymphoma and leukemia, except malignant neoplasm of skin ** or Metastatic solid tumour.

For peer review only

Supplementary Table 5. Triage at hospital (RETTS and NEWS 2) and contact cause at hospital

**Triage**	**Non-survivors, n=195**	**Survivors, n=195**	**p-value**
RETTS Red (%)	76 (46) n164	62 (35) n177	*0.03*
RETTS Orange (%)	51 (31) n164	71 (40) n177	0.08
RETTS Yellow (%)	32 (20) n164	42 (24) n177	0.35
RETTS Green (%)	5 (3) n164	2 (1) n177	0.27
RETTS Red plus Orange (%)	127 (77) n164	133 (75) n177	0.62
NEWS 2 score (SD)	7.1 (4.0) n163	5.0 (3.4) n181	*<0.001*
NEWS 2 score ≥5 (%)	114 (70) n163	89 (49) n181	*<0.001*
**Reason for first hospital contact (number) (%)**			
Fever	86 (44)	119 (61)	*<0.01*
Chills	26 (13)	50 (26)	*0.02*
"Found on the floor"	18 (9)	5 (3)	*0.01*
Gastrointestinal symptoms	32 (16)	32 (16)	>0.99
Cough	8 (4)	15 (8)	0.13
Dyspnea/breathing difficulties	60 (31)	46 (24)	0.11
Urinary tract symptoms	13 (7)	25 (13)	*0.04*
Rapid deterioration in general condition	81 (42)	48 (25)	*<0.01*
Fatigue	14 (7)	17 (9)	0.57

Data are presented as no. (%) or mean (SD). Pearson chi2, Fisher's exact test or T-test, as appropriate. RETTS: Red = life-threatening condition, Orange = potentially life-threatening. Yellow/Green = acute process can wait.

For peer review only

STROBE Statement—Checklist of items that should be included in reports of case-control studies

	Item No	Page No
**Titel and abstract**	1	1
**Introduction**		
Background	2	3-4
Objectives	3	4
**Methods**		
Study design	4	4
Setting	5	4
Participants	6	4-5
Variables	7	4-7
Data sources	8	4-6
Bias	9	N/A
Study size	10	4-5
Quantitative variables	11	8
Statistical methods	12	8
**Results**		
Participants	13	9, Flow-chart
Descriptive data	14	9, table 1,2 and supplementary files
Outcome data	15	9-11, Table 1, 2
Main results	16	11-12
Other analyses	17	N/A
**Discussion**		
Key results	18	12
Limitations	19	15-16
Interpretation	20	12-15, 16
Generalisability	21	12-15, 16
**Other information**		
Funding	22	16

For peer review only

Prehospital delay is an important risk factor for mortality in community-acquired bloodstream infection (CA-BSI): A matched case-control study

Journal:	*BMJ Open*
Manuscript ID	bmjopen-2021-052582.R1
Article Type:	Original research
Date Submitted by the Author:	01-Sep-2021

Complete List of Authors:	Holmbom, Martin; Linköping University, Department of Biomedical and Clinical Sciences; Linköping University, Department of Urology<br>Andersson, Maria; Linköping University, Division of Infectious Diseases, Department of Biomedical and Clinical Sciences, Faculty of Medicine and Health Sciences, Linköping University, Linköping, Sweden<br>Berg, Sören; Unit for Cardiovascular Sciences, Faculty of Medicine and Health Sciences<br>Eklund, Dan; Linköping University, Division of Infectious Diseases, Department of Biomedical and Clinical Sciences, Faculty of Medicine and Health Sciences, Linköping University, Linköping, Sweden<br>Sobczynski, Pernilla; Linköping University, Division of Infectious Diseases, Department of Biomedical and Clinical Sciences, Faculty of Medicine and Health Sciences, Linköping University, Linköping, Sweden<br>Wilhelms, Daniel; Linköping University, Department of Health and Medical Sciences,; Local Health Care Services in Central Östergötland, Department of Emergency Medicine<br>Moberg, Anna; Linköping University, Department of Health, Medicine and Caring Sciences, Linkoping University, Linkoping, Sweden<br>Fredrikson, Mats; Department of Clinical and Experimental Medicine, Faculty of Health Sciences<br>Balkhed, Åse Östholm; Linköping University, Division of Infectious Diseases, Department of Biomedical and Clinical Sciences, Faculty of Medicine and Health Sciences, Linköping University, Linköping, Sweden<br>Hanberger, Håkan ; Linköping University

**Primary Subject Heading**:	Infectious diseases
Secondary Subject Heading:	Emergency medicine, General practice / Family practice
Keywords:	Adult intensive & critical care < INTENSIVE & CRITICAL CARE, ACCIDENT & EMERGENCY MEDICINE, Public health < INFECTIOUS DISEASES, INFECTIOUS DISEASES, PRIMARY CARE

SCHOLARONE™
Manuscripts

I, the Submitting Author has the right to grant and does grant on behalf of all authors of the Work (as defined in the below author licence), an exclusive licence and/or a non-exclusive licence for contributions from authors who are: i) UK Crown employees; ii) where BMJ has agreed a CC-BY licence shall apply, and/or iii) in accordance with the terms applicable for US Federal Government officers or employees acting as part of their official duties; on a worldwide, perpetual, irrevocable, royalty-free basis to BMJ Publishing Group Ltd ("BMJ") its licensees and where the relevant Journal is co-owned by BMJ to the co-owners of the Journal, to publish the Work in this journal and any other BMJ products and to exploit all rights, as set out in our licence.

The Submitting Author accepts and understands that any supply made under these terms is made by BMJ to the Submitting Author unless you are acting as an employee on behalf of your employer or a postgraduate student of an affiliated institution which is paying any applicable article publishing charge ("APC") for Open Access articles. Where the Submitting Author wishes to make the Work available on an Open Access basis (and intends to pay the relevant APC), the terms of reuse of such Open Access shall be governed by a Creative Commons licence – details of these licences and which Creative Commons licence will apply to this Work are set out in our licence referred to above.

Other than as permitted in any relevant BMJ Author's Self Archiving Policies, I confirm this Work has not been accepted for publication elsewhere, is not being considered for publication elsewhere and does not duplicate material already published. I confirm all authors consent to publication of this Work and authorise the granting of this licence.

For peer review only

Prehospital delay is an important risk factor for mortality in community-acquired bloodstream infection (CA-BSI): A matched case-control study

Martin Holmbom[1,2], Maria Andersson[1], Sören Berg[3], Dan Eklund[1], Pernilla Sobczynski[1], Daniel Wilhelms[4], Anna Moberg[5], Mats Fredrikson[6], Åse Östholm Balkhed[1*], Håkan Hanberger[1*]

1 Division of Infectious Diseases, Department of Biomedical and Clinical Sciences, Faculty of Medicine and Health Sciences, Linköping University, Linköping, Sweden, **2** Department of Urology and Department of Biomedical and Clinical Sciences, Linköping University, Linköping, Sweden, **3** Unit for Cardiovascular Sciences, Faculty of Medicine and Health Sciences, **4** Department of Emergency Medicine in Linköping, and Department of Biomedical and Clinical Sciences, Linköping University, Linköping, Sweden **5** Department of Health, Medicine and Caring Sciences, Linkoping University, Linkoping, Sweden, **6** Department of Biomedical and Clinical Sciences and Forum Östergötland, Faculty of Medicine and Health Sciences. * Contributing equally to this work.

Corresponding author: Martin Holmbom, martin.holmbom@regionostergotland.se

ABSTRACT

Objectives: The aim of this study was to identify prehospital and early hospital risk factors associated with 30-day mortality in patients with blood culture-confirmed community-acquired bloodstream infection (CA-BSI) in Sweden.

Methods: A retrospective case-control study of 1,624 patients with CA-BSI (2015-2016), 195 non-survivors satisfying the inclusion criteria were matched 1:1 with 195 survivors for age, gender, and microorganism. All forms of contact with a healthcare provider for symptoms of infection within 7 days prior CA-BSI episode were registered. Logistic regression was used to analyse risk factors for 30-day all-cause mortality.

Results: Of the 390 patients, 61% (115 non-survivors and 121 survivors) sought prehospital contact. The median time from first prehospital contact till hospital admission was 13 hours (6-52) for non-survivors and 7 hours (3-24) for survivors ($p<0.01$). Several risk factors for 30-day all-cause mortality were identified: prehospital delay OR=1.26 (CI: 1.07-1.47), $p<0.01$; severity of illness (SOFA-score) OR=1.60 (CI: 1.40-1.83), $p<0.01$; comorbidity score (updated Charlson Index) OR=1.13 (CI: 1.05-1.22), $p<0.01$; and inadequate empirical antimicrobial therapy OR=3.92 (CI: 1.64-9.33), $p<0.01$. In a multivariable model, prehospital delay >24 hours from first contact

For peer review only

remained an important risk factor for 30-day all-cause mortality due to CA-BSI OR=6.17 (CI: 2.19-17.38), $p<0.01$.

Conclusion: Prehospital delay and inappropriate empirical antibiotic therapy were found to be important risk factors for 30-day all-cause mortality associated with CA-BSI. Increased awareness and earlier detection of bloodstream infection in prehospital and early hospital care is critical for rapid initiation of adequate management and antibiotic treatment.

Article summary

Strengths and limitations of this study

- Non-surviving patients were matched 1:1 with survivors for age, gender, and microorganism to evaluate risk factors for 30-day mortality.
- Data on limitation of level of care were collected and evaluated as a potential confounding factor.
- Only empirical antibiotic treatments were analysed, early changes in therapy were not considered.
- Data regarding patient delay *i.e.,* time from onset of symptoms to contact with prehospital or hospital care, were unavailable.

For peer review only

Background

Bloodstream infection (BSI) is a major cause of morbidity and mortality worldwide, with sepsis and septic shock as important complications [1-5]. In Europe, the number of BSIs and number of deaths due to BSI each year have been estimated to be 1.2 million and 157 000 respectively [6]. Most studies in Nordic countries report an increase in the incidence of BSI [7-10] and an increase in associated mortality [7, 9], but a low level of antibiotic resistance [11].

The increasing incidence of community-onset bloodstream infection has become a major problem. This may be due to an increase in healthcare-associated BSI related to complex medical care of aged patients with comorbidity in the community setting [7, 12]. BSI and the risk of transition to sepsis and septic shock with life-threatening organ dysfunction, requires early identification for prompt initiation of life-saving measures and improvement in outcome [13-17]. There are many studies focusing on improving early detection of patients at risk for developing sepsis in the Emergency Department (ED) [18-22]. Few studies have addressed prehospital care of sepsis prior to evaluation at the ED or by the paramedic [23, 24], and not one, to our knowledge, on the impact of delay in prehospital care on 30-day mortality in a well-defined case-control study on patients with community-acquired bloodstream infection (CA-BSI). The aim of this study was to identify prehospital and early hospital risk factors associated with 30-day mortality in patients with culture-confirmed CA-BSI

For peer review only

METHODS

Study design and setting

This was a case-control study on risk factors for 30-day mortality in patients with CA-BSI. From a database of culture-confirmed CA-BSI in the Swedish county of Östergötland, samples of cases (non-survivors) and matched controls (survivors) over a two-year period (from 1st January 2015 until 31st December 2016) were selected. The county of Östergötland, with a population of 445,000 in December 2015 and 452,000 in December 2016, had four hospitals, one tertiary care university hospital, two general hospitals, one district hospital, 44 primary healthcare centres (PHCCs), and four out-of-office hours centres.

In most West European countries, prehospital healthcare is available at several levels. In this study, prehospital care is defined as healthcare contact prior to transport by ambulance or entering the Emergency Department (ED). In Sweden, first contact with the healthcare service is usually by a phone call to the PHCC or to the National Healthcare Guide (NHG). In urgent situations, the emergency medical service (*i.e.*, paramedic ambulance service) is contacted directly. PHCCs usually provide a call-back system where a nurse rings during office hours and assesses the need for clinical assessment. The NHG offers immediate medical advice over the phone twenty-four hours a day. It is manned by specially trained nurses using a decision-support algorithm, they assess the need for further care and provide advice and/or recommend other healthcare services.

Data collection

From the microbiology laboratory database, the following dataset was collected: blood culture results, number of aerobic and anaerobic blood culture vials taken, sites of puncture; species identification, and susceptibility patterns. The dataset was entered into a second database where it

For peer review only

106 was linked to the patient-administration system providing the following data on all patients with a BSI episode: gender, age, comorbidity, admitting department, date of admission, date of discharge, and mortality.

109 A total of 2,356 BSI episodes were identified, of which 1,624 were CA-BSIs. To determine the impact of prehospital and early hospital care on 30-day all-cause mortality 2015-2016, we matched a group of non-survivors (cases) with survivors (controls) that satisfied the following inclusion criteria: adult (≥ 18 years), culture-confirmed CA-BSI with a significant pathogen, registered in the County of Östergötland, treated in a hospital in the County of Östergötland, non-survivor (dead within 30 days from positive blood culture) and survivor alive at (30 days).

115 In all, 195 non-survivors met the inclusion criteria and were matched with 195 survivors for age (+/- 10 years), gender, and microorganisms (Fig 1).

117

118 For non-survivors and survivors data on all infection-related contacts with a healthcare provider (telephone contact or physical contact) ≤7 days prior to a positive blood culture were collected by reviewing medical records. For each contact, the following were documented in the Case Report Form: profession of healthcare provider, suspected diagnosis, vital signs, primary focus of infection, and initial advice given. In the event of ambulance transport, information on time to reach the patient, time to reach ED, vital signs, intravenous fluid administration, and oxygen administration were extracted from the mission report.

125 The following data on management in the ED were collected: limitation of level of care, medical personnel involved, vital signs, laboratory data, consultation with a specialist if any (infectious diseases, internal medicine, anaesthesia *etc.*), time on the ED, times to fluid administration and antibiotic treatment, other sepsis treatment such as corticosteroids, and highest serum lactate within 24 hours after admission. The highest priority given by the triage system RETTS (Rapid Emergency

For peer review only

Triage and Treatment System) was documented [25, 26]. Sequential Organ Failure Assessment (SOFA) score [27] and National Early Warning Score (NEWS) 2 [28] were also calculated.

An expert group consisting of physicians specialised in infectious diseases judged the correctness of the antibiotic treatment based on local recommendations for empirical treatment of the suspected focus at that time, and the pathogen finally cultured. Doses of antibiotics, corrected for renal function, were also judged. Comorbidity scores according to the modified Charlson Comorbidity Index, based on diagnoses obtained from patient medical records up to 24 months prior to the BSI, were later entered into the database.

For peer review only

Definitions

Blood culture: One set of blood cultures comprised one aerobic and one anaerobic blood culture bottle. It was recommended that at least two sets of blood cultures be taken simultaneously.

Positive blood culture: The isolations of microorganism/s (one or more bacterial or fungal isolates) from a set of blood cultures. Only bacterial or yeast isolates from initial blood cultures were considered; repeat isolates were excluded.

Non-significant pathogens: Microorganisms typically belonging to the skin microbiome (Coagulase-Negative Staphylococci, (CoNS), *Micrococcus* spp., *Bacillus* spp., *Corynebacterium* spp. and *Propionibacterium* spp.) were considered probable contaminants and excluded [29]. An exception was CoNS isolated from blood cultures taken from at least two different puncture sites on the same occasion, and with an identical resistance pattern.

Community-acquired bloodstream infection (CA-BSI): A positive blood culture obtained within 48 hours after hospital admission. Cultures from readmission within 30 days were excluded. Furthermore, patients who had received intravenous treatment or advanced nursing care at home such as parenteral nutrition, haemodialysis, or intravenous chemotherapy, within the previous 30 days were excluded.

155 **Immunosupression:** Patients were deemed immunocompromised if prescribed immunosuppressive medication or had a code number (D80-D89) for an immunosuppressive disease according to the International Classification of Diseases, 10th edition (ICD-10) when admitted to the hospital.

158 **Comorbidity:** Comorbidity score was based on the updated Charlson Comorbidity Index [30-32] using ICD-10 diagnosis codes documented in the patient's medical record up to 24 months prior to date of admission (Supplementary File, Table 1).

161 **Sepsis:** The Sepsis-3 definition [33] was used *i.e.*, an increase by 2 points or more in the SOFA score due to infection.

163 **Limitation of level of care:** In some cases, the decision was made to limit the level of care. Such a decision influences treatment provided and thus mortality. This is an obvious confounding factor. Limitation of level of care set in the ED or within the first 24 h of admission was registered as: (a) withholding of intensive care (no ICU); (b) withholding of cardiopulmonary resuscitation (no CPR); and (c) withdrawal of all active treatment *i.e.*, end-of-life care (EoLC).

168 **Appropriate empirical antibiotic therapy**: Defined by local recommendations, suspected source of infection, severity of illness, dosage, and correction for renal function [34, 35].

170 **Microbiologically appropriate empirical antibiotic therapy**: Defined according to cultured pathogen and its susceptibility patterns [34-36].

For peer review only

Microbiological methods

All microorganisms isolated were analysed at the species level. Microorganism identification and susceptibility testing were performed at the Clinical Microbiology Department, University Hospital, Linköping using matrix-assisted laser desorption ionisation time-of-flight mass spectrometry (MALDI-TOF MS). Antibiotic susceptibility classification used during study period was: Susceptible (S), Intermediate (I), and Resistant (R), according to the European Committee on Antimicrobial Susceptibility Testing (EUCAST) guidelines [36].

Statistical analysis

Data are presented as mean and standard deviation (SD) or median and quartiles (Q1-Q3). We compared normally distributed quantitative variables using the Student *t* test and categorical variables using the chi^2 test or Fisher exact test as appropriate. Mann–Whitney U test and Kruskal Wallis test were used otherwise. A two-tailed p value < 0.05 was considered statistically significant. Correlations were analysed according to Pearson. Missing data were treated by pairwise deletion, and there was no imputation. Univariate and multivariable analyses were performed with logistic regression including the descriptive parameters that correlated to 30-day mortality. Two patients among the non-survivors had EoLC taken prior to admission to the hospital. This was considered to have had a major effect on early treatment and time to admission to hospital for these patients and they and their matched survivors were excluded from the analyses regarding time to admission and the multiple regression model. The statistics programme SPSS version 25 was used for the analyses.

For peer review only

Results

Demographic and clinical characteristics

A total of 2,356 BSI episodes were identified during the study period, of which 1,624 (69%) were CA-BSIs. The all-cause 30-day mortality rate of CA-BSI was 12.6% (n=205). Of these, 95% (n=195) met the inclusion criteria and were included in the study as non-survivors (case-group) and were matched with 195 survivors (control group) for gender, age, and microorganism. Almost 40% of the non-survivors died within 3 days after admission, and 80% within 7 days. Underlying comorbidity scores according to the updated Charlson Comorbidity Index were higher among non-survivors than survivors (3.2 vs 2.3, p=<0.01). Non-survivors were more often diagnosed with cancer (34% vs 23%, p=0.01) and metastatic carcinoma (14% vs 7%, p=0.02) compared to survivors. Limitation of level of care before admission was significantly more common among non-survivors (14% vs 2%, p=<0.01), (Table 1 and Supplementary File, Table 2).

Microbiology

There were 407 isolates from 390 cases. *Staphylococcus aureus* was the most frequently isolated primary pathogen (27%), followed by *Escherichia coli* (21%), *Streptococcus pneumoniae* (8%) and *Klebsiella pneumoniae* (7%). *Candida* spp. were observed in 2% of cases. Polymicrobial CA-BSI was found in 9 non-survivors and 8 survivors. Microbiological findings are summarised in Supplementary File, Table 3.

For peer review only

Table 1. Demographics and prehospital data

	Non-survivors n=195	Survivors n=195	p-value
**Demographics (%)**			
Male	106 (54)	106 (54)	>0.99
Mean age (SD)	78y (±13)	76y (±12)	0.20
Surgery within 30d	29 (15)	27 (14)	0.77
Immunosuppression	60 (31)	45 (23)	0.09
**Charlson (update weight) (SD)**	3.2 (2.99)	2.3 (2.36)	*0.01*
**Patients with any limitation of level of care before admission (%)**	28 (14)	4 (2)	*<0.01*
EoLC	2 (1)	0	0.50
No ICU	17 (9)	2 (1)	*<0.01*
No CPR	27 (14)	4 (2)	*<0.01*
**First prehospital contact site (%)**			
Phone to NHG	14 (7)	60 (31)	*<0.01*
Phone to a PHCC	79 (41)	33 (17)	*<0.01*
Patient visit to a PHCC	22 (11)	28 (14)	0.36
No prehospital contact	80 (41)	74 (38)	0.53
**Multiple prehospital contacts (≥2) (%)**	64 (33)	41 (21)	*0.01*
**Reason for first prehospital contact (number) (%)**	n115	n121	
Fever	44 (38)	43 (36)	0.67
Chills	13 (11)	13 (11)	0.89
"Found on the floor"	4 (3)	0	*0.04*
Gastrointestinal symptoms	16 (14)	23 (19)	0.29
Cough	3 (3)	13 (11)	*0.01*
Dyspnoea/breathing difficulties	23 (20)	15 (12)	0.11
Urinary tract symptoms	14 (12)	22 (18)	0.20
Rapid deterioration in general condition	47 (41)	29 (24)	*<0.01*
Fatigue	3 (3)	15 (12)	*<0.01*
**Time from first prehospital contact to admission, hours, median (Q1-Q3)**	13 (5.9-51.6) n113	7.2 (3.3-24) n119	*<0.01*
Phone to NHG	4 (1.0-8.7) n13	3.6 (1.3-7.3) n58	0.64
Phone to a PHCC	24 (6.8-72) n78	12.5 (5.9-24) n33	0.06
Visit to a PHCC	11 (6.6-77) n22	12.5 (6.4-66) n28	0.75
**Time from first prehospital contact to admission, intervals (%)**	n193	n193	
0-6 hours	29 (15)	49 (25)	*0.01*
6-12 hours	26 (14)	30 (16)	0.56
12-24 hours	12 (6)	21 (11)	0.10
>24 hours	46 (24)	19 (10)	*<0.01*
No prehospital contact	80 (41)	74 (38)	0.53

Data are presented as no. (%) or mean (SD). Pearson chi2, Fisher's exact test or T-test, as appropriate. P values < 0.05 are shown in italics. Time indications are calculated with median, interquartile 25th to 75th percentile range (Q1-Q3) and Mann–Whitney U. Reason for first prehospital contact: more than one reason possible. NHG = national healthcare guide. PHCC=primary healthcare centre.

For peer review only

Prehospital data

Of the 390 patients, 61% (115 non-survivors and 121 survivors) had taken contact with a prehospital healthcare facility for infection-related symptoms within 7 days prior to admission to hospital. Forty-four per cent of these patients had ≥2 prehospital contacts. Forms of prehospital contact were phone contact with a PHCC (47%), phone contact with NHG (31%), and visit to a PHCC (21%). Fever was the primary reason for prehospital contact (37%). Rapid deterioration in general condition was the second most common reason (32%) and was more common among non-survivors than survivors (41% vs 24%, p<0.02) (Table 1). Phone contact with a PHCC was the more common form of first contact among non-survivors (41% non-survivors vs 17% survivors, p=0.01), whereas phone contact with NHG was more common among survivors (7% non-survivors vs 31% survivors, p=<0.01) (Table 1).

Time from first infection-related prehospital contact till hospital admission

In total, the median time from first prehospital contact till hospital admission was 8 hours (4-27). This was significantly longer for non-survivors 13 hours (6-52) vs 7.2 hours (3-24) for survivors, p=0.01 (Table 1). For the study group as a whole, there were significant differences in time to admission depending on form of first contact, with shorter median time to admission after telephone contact with the NHG 3.8 hours (1.3-7.7) compared to phone contact with a PHCC 24.0 hours (6.8-48) or visit to a PHCC 11.3 hours (6.7-72) (p<0.01).

For peer review only

Hospital data

A total of 88% were admitted to hospital via the ED (85% non-survivors vs 91% survivors, p=0.04), other patients were admitted via a specialist outpatient department. Hospital admission after ambulance transport was more common among non-survivors (81% vs 70%, p=0.01) (Table 2). The most common sign reported when admitted to hospital was fever (54%). The most common infection focus was a urinary tract infection (24%) followed by infection with unknown focus (23%), and respiratory tract infection (22%). There were no differences between non-survivors and survivors regarding primary focus of infection (Supplementary File, Table 4).

On the ED, vital signs and most laboratory test results indicated a more serious condition in non-survivors compared to survivors and 159 non-survivors and 161 survivors were triaged with a life-threatening or potentially life-threatening condition according to the RETTS score. Abnormalities in respiratory function, haemodynamics, and neurological function were significantly more prominent among non-survivors (Supplementary File, Table 4). SOFA score on admission and at 24 hours was higher among non-survivors 4.2 (SD: 2.3) vs 2.3 (SD: 1.7), (p<0.01) and 6.8 (3.6) vs 3.8 (2.7) (p<0.01) respectively). On admission to hospital, 75% of non-survivors and 52% of survivors (p<0.01) fulfilled the Sepsis 3 criteria, and within 24 hours the corresponding figures were 95% of non-survivors and 79% of survivors (p<0.01). In all, 11% of non-survivors received ICU care within 24 hours compared to 5% of survivors (p=0.01) (Table 2).

For peer review only

Table 2. Hospital data - Severity of diseases and antibiotic treatment

	Non-survivors n=195	Survivors n=195	p-value
**Ambulance transport (%)**	158 (81) n194	136 (70) n193	*0.01*
**Admission to hospital (%)**			
Emergency department	165 (85)	178 (91)	*0.04*
Specialist outpatient department	30 (15)	17 (9)	*0.04*
**Severity of disease (SD)**			
Habitual SOFA	0.8 (1.1) n185	0.4 (0.8) n193	*<0.01*
SOFA score on admission	4.2 (2.3) n161	2.3 (1.7) n160	*<0.01*
SOFA score at 24 h	6.8 (3.6) n 187	3.8 (2.7) n 195	*<0.01*
Maximum lactate level mmol/l first 24 hr	4.6 (4.0) n 122	2.9 (1.9) n 110	*<0.01*
NEWS 2	7.1 (4.0) n 163	5.0 (3.4) n 181	*<0.01*
**Sepsis (%)**			
Sepsis on admission	119 (75) n158	82 (52) n158	*<0.01*
Sepsis at 24 h	176 (95) n185	152 (79) n193	*<0.01*
**ICU care within 24 hours (%)**	22 (11.3)	9 (4.6)	*0.01*
**Antibiotic treatment**			
Antibiotic administration (%)	186 (96) n193	192 (99.5) n193	0.07
Time (hours) to antibiotics from admission median, (Q1-Q3)	2.8 (1.4-5.3) n185	3.0 (1.4-6.2) n192	0.58
First dose antibiotic within one hour (%)	34 (18) n185	30 (16) n192	0.48
Appropriate empirical antibiotic therapy (%)	162 (87) n186	185 (96) n192	*<0.01*
Microbiologically appropriate empirical antibiotic therapy (%)	155 (83) n186	168 (88) n192	0.25
**Intravenous fluids in the ED (%)**	124 (76) n163	116 (68) n171	0.09

Data are presented as no. (%) or mean (SD). Pearson chi2, Fisher's exact test or T-test, as appropriate. P values < 0.05 are shown in italics. Time indications are calculated with median, interquartile 25th to 75th percentile range (Q1-Q3) and Mann–Whitney. Two non-survivors with end-of-life care (EoLC) on arrival at hospital, and their controls were excluded from the analysis regarding antibiotic treatment and intravenous fluids

Antibiotic treatment was initiated in 96% of non-survivors and 99.5% of survivors, (p=0.07). The median time from hospital admission to start of empirical antibiotic did not differ between the groups, non-survivors 2.8 hours (1.4-5.3) and survivors 3.0 hours (1.4-6.2), nor in the case of sub-classification according to triage score (RETTS) (Supplementary File, Table 5). Antibiotic administration was started within one hour in 18% of non-survivors and 16% of survivors (p=0.48). More survivors received appropriate empirical antibiotic therapy than non-survivors (87% non-survivors and 96% survivors, p<0.01). Microbiologically appropriate empirical antibiotic therapy did not differ significantly between the groups (82% non-survivors vs 88% survivors) (Table 2). The empirical antibiotics used are listed in Supplementary File, Table 6.

For peer review only

Analysis of risk factors for 30-day mortality in CA-BSI

In the univariate logistic regression several risk factors associated with 30-day mortality were observed: comorbidity score (updated Charlson Index) OR = 1.13 (CI 1.05-1.22), p<0.01, prehospital delay OR = 1.26 (CI 1.07-1.47), p=0.01, severity of illness (SOFA score on admission) OR = 1.60 (CI 1.40-1.83), p<0.01 and inappropriate empirical antibiotic therapy OR = 3.92 (CI 1.64-9.33), p<0.01 (Table 3).

In the multivariable model of 30-day mortality, prehospital delay >24 hours was significantly related to mortality, OR = 6.17 (CI 2.19-17.38), p<0.01. This was followed by inappropriate empirical antibiotic therapy, OR = 5.50 (CI 1.62-18.63), p=0.01, no prehospital contact, OR = 2.56 (CI 1.02-6.41), p=0.045, and severity of illness (SOFA score on admission), OR = 1.35 (CI 1.16-1.57), p<0.01 (Table 3).

Table 3. Risk factors for 30-day mortality

	**Univariate analysis**			**Multivariable analysis**		
**Risk factor**	**OR**	**95% CI**	**p-value**	**OR***	**95% CI**	**p-value**
**Time from first prehospital contact to admission (intervals)**	1.26	1.07-1.47	*0.01*			
0-6h	1**			1**		
6-12h	1.46	0.73-2.94	0.28	1.35	0.48-3.79	0.56
12-24h	0.97	0.42-2.25	0.94	0.56	0.15-2.10	0.39
>24h	4.09	2.02-8.28	*<0.01*	6.17	2.19-17.38	*<0.01*
No prehospital contact	1.83	1.05-3.19	*0.03*	2.56	1.02-6.41	0.05
**Updated Charlson**	1.13	1.05-1.22	*<0.01*	1.02	0.91-1.15	0.70
**Ambulance transport**	1.84	1.14-2.97	*0.01*	1.59	0.68-3.71	0.29
**Admission through ED**	0.55	0.29-1.03	0.06	1.35	0.43-4.25	0.61
**ln SOFA score**	1.60	1.40-1.83	*<0.01*	1.35	1.16-1.57	*<0.01*
**Inappropriate empirical antibiotic therapy*** **	3.92	1.64-9.33	*<0.01*	5.50	1.62-18.63	*0.01*
**Rapid deterioration in general condition**	1.79	1.06-3.02	*0.03*	2.27	0.98-5.28	0.06
**Any care restrictions before or within 24h after admission**	9.95	5.95-16.64	*<0.01*	10.37	4.96-21.67	*<0.01*
**Matching**	1	0.99-1.00	>0.99	1	0.99-1.0	0.20

** Multivariable binomial regression analysis. ** Reference. *** Clinical inappropriateness defined by local recommendations, suspected source of infection, severity of illness, dosage, and correction for renal function.*

For peer review only

Discussion

This study revealed that prehospital delay had a major impact on mortality among patients with CA-BSI. Non-survivors were more seriously ill when admitted to hospital, with significantly higher SOFA scores and more signs of sepsis. Although initial care at the hospital and time to antibiotic treatment were comparable between non-survivors and survivors (for some variables even better among non-survivors) this was not enough to compensate for the negative effect of prehospital delay.

CA-BSI patients form a heterogeneous group in which underlying disease, gender, age, and appropriate management vary significantly. The severity of bloodstream infection ranges from asymptomatic bacteraemia to fulminant sepsis or septic shock. To our knowledge, this is the first study to focus on a well-defined population of adult patients with CA-BSI focusing on prehospital care prior to hospital admission. In previous study by Holmbom *et al*, gender, age, and pathogen were risk factors for mortality in BSI [7, 11], so these were used when matching patients to enable evaluation of other potential risk factors in this study. Compared to previous studies and reports on sepsis and CA-BSI, the distribution of pathogens in the present study was different [7, 37, 38], with *S.aureus* being the most common. This was probably a result of the study design *i.e.,* inclusion of patients dying within 30 days and matched controls, since *S.aureus* BSI has such a high mortality rate [39, 40].

Delay in antibiotic treatment has previously been shown to have a clear association with increased mortality in progressive sepsis and septic shock [13, 15, 17, 37, 41, 42]. However, delay prior to ambulance transport or coming to the ED, and causes of delay have hardly been investigated [24]. Strategies for early identification of sepsis in prehospital care, and measures to increase survival from sepsis have received much attention, and several strategies and screening tools have been investigated with varied results [18, 21, 43]. Among the patients in the present study who took prehospital contact with the healthcare service, form of first prehospital contact affected prehospital

For peer review only

delay. Telephone contact with the NHG was associated with shorter time to hospital admission than phone contact or visit to the local PHCC. The reason for this remains unclear. Possible factors include: 1. Compliance to the NHG computer-based decision algorithm designed for acute illness, as well as nurses more experienced in giving advice over the phone; 2. More liberal attitude of NHG nurses to send patients to the ED; 3. Non-survivors have higher comorbidity scores and tend to seek initial contact with their PHCC. Frail patients have also greater difficulty in describing their symptoms, making it more difficult for the primary healthcare provider to evaluate the seriousness of their condition; and 4. Whereas the NHG gives advice directly, it is possible for the PHCC to delay clinical assessment until later the same or next day. In fact, the call-you-back system used by PHCCs may have caused even more delay than seen in this study since we did not have access to when the first phone call from the patient was made. Instead, we used the time the phone-call back to the patient was made. In conclusion, the BSI healthcare process and its outcome varies according to the form of healthcare provider the patient first contacts.

We also looked for factors facilitating earlier identification of patients with increased risk for death. The comorbidity score (updated Charlson Comorbidity Index) was higher among non-survivors, with higher rates of cancer and metastatic carcinoma. These conditions are associated not only with increased mortality risk in general but also with BSI itself [7, 11, 44]. In prehospital records, “found on the floor” and “rapid deterioration in general condition” were more often reported among non-survivors. Since both reflect a seriously ill person, the patient is by proxy identified as a patient at risk. Although early prehospital identification of patients at high risk for sepsis or mortality is complex [43], we confirm the findings of previous studies [7, 11, 44] that taking signs and symptoms of infection in cancer patients seriously is crucial. Otherwise, we found no patient-specific factors that could be of help in identifying patients with increased mortality risk.

For peer review only

Many studies on sepsis, especially prior to Sepsis 3, have had patients with a wide spectrum of disease severity. This has certainly contributed to differences in results regarding the impact of time to antibiotic treatment on outcome [13-15, 17]. Our study comprised patients with CA-BSI, not true sepsis, and thus even more likely to be heterogeneous. Even so, 75% of non-survivors and 52% of survivors had sepsis on admission to the ED. The severity of illness on admission to hospital was greater in non-survivors and there are several factors that might explain this. One factor could be a longer prehospital delay as observed in this study.

Previous studies have shown the importance of rapid detection and initiation of adequate antibiotic treatment in sepsis and especially septic shock [13-15, 17, 37]. Nevertheless, using the RETTS triage system [25, 33], only half of the non-survivors were captured by the highest priority (red). Furthermore, priorities red plus orange together would still have failed to detect one third. Likewise, when using NEWS 2 with the proposed cut-off score of 5 [28, 45], at least one third of the non-survivors would have remained undetected as patients at risk for sepsis in the initial triage. Triage is useful when escalating the care of patients already identified with sepsis, as shown by Rosenqvist *et al* [22], but we lack a valid, simple, highly predictive scoring system to detect sepsis [46, 47]. Surviving Sepsis Guidelines 2016 recommend that antibiotics be administered within one hour of diagnosing sepsis [48]. This was not achieved in our material where only 16% of non-survivors and 18% of survivors received the first dose of antibiotics within one hour. Appropriate empirical antibiotic therapy according to local guidelines was more common among survivors ($p<0.01$), while microbiologically appropriate empirical antibiotic therapy was not. This could indicate that not only correct empirical antibiotics is important, but that also a holistic view of the patient, including suspected focus of infection and need of source control, are important parts of early management. This study showed that, prehospital delay and non-appropriate empirical treatment among patients with CA-BSI worsens the outcome and measures should be taken both pre-hospital and at the emergency unit to increase survival in CA-BSI.

For peer review only

Limitations

To our knowledge, this is the first study on a well-defined population of adult patients with CA-BSI, focusing on prehospital care prior to hospital admission, but it has limitations.

1. Though non-survivors and survivors were matched for gender, age (+/- 10 years), and microorganism, microorganism resistance patterns were not considered when matching non-survivors and survivors. However, since the prevalence of resistant bacteria in Sweden is low, this could only have had a minor influence on the results. Furthermore, when analysing correct antimicrobial therapy based on microorganisms cultured and their resistance patterns, there was no significant difference between the groups.
2. We only analysed the correctness of empirical antibiotic treatment, while early changes in therapy were not considered.
3. Other causes of death such as myocardial infarction, respiratory failure or pulmonary embolism as primary cause of death were possible, but such conditions would likely be related to the underlying infection, and since 95% of the cases had a fulminant sepsis within 24 hours this would be the major predisposing factor.
4. The only differences in prehospital management affecting outcome in this study were the timing of events. Other variables require further research.
5. The only time parameters for early hospital care in this study were time to empirical antibiotic treatment and time for ambulance transport. Presumably, time to antibiotic treatment also implies time to early hospital treatment. Furthermore, time to early hospital care is usually based on severity of illness (RETTS and NEWS 2) when admitted to hospital. Time to antibiotic treatment based on RETTS did not differ between the groups. This study was not designed to validate the time to care based on triage level, RETTS or NEWS 2 correctness.

For peer review only

6. Community healthcare in Sweden has several healthcare providers i.e., care provided by the community, private medicine and/or self-medication (by patient or relative). This result in unavailable data, and it is possible that some community-acquired healthcare-associated BSIs may have occurred in the study population, even though hospital and healthcare-related infections were excluded as far as possible.
7. Data regarding patient delay *i.e.,* time from onset of symptoms to contact with prehospital or hospital care should be included when calculating hospital and therapy delay. However, these were unavailable since patient delay was not systematically registered in the patient records.

Conclusion

In this case-control study, prehospital delay and inappropriate empirical antibiotic therapy were found to be important risk factors for 30-day all-cause mortality associated with CA-BSI. Effective guidelines to aid recognition of patients likely to develop sepsis in prehospital and early hospital care, and an effective prehospital medical advice service, could help to make significant progress in the care of patients with bloodstream infection.

Acknowledgements

Thanks to consultant anaesthetist Peter Cox for proof reading.

Competing interests

None declared.

Author Contributions

MH, HH and ÅÖB conceived and designed the study. MH, MA, DE, ÅÖB and MF analysed the data. MH and MA wrote the manuscript. All authors (MH, MA, SB, DE, PS, DW, AM, MF, ÅÖB

For peer review only

and HH) contributed to the discussion and reviewed the manuscript. All authors (MH, MA, SB, DE, PS, DW, AM, MF, ÅÖB and HH) commented and approved the final version of the paper.

Data Availability

All relevant data are within the manuscript and its Supplementary File.

Funding

This work was supported by Östergötland Count Council (Award/grant number: N/A).

Patient and public involvement

There was no patient and/or public involvement in the design of the study.

Ethics approval

The Regional Ethics Review Board in Linköping, Sweden, approved the study (Ref. no: 2010/160-31).

Figure legends

Fig 1. *A total of 2,356 BSI episodes were identified, of which 1,624 were CA-BSIs, 195 non-survivors met the inclusion criteria and were matched with 195 survivors for age (+/- 10 years), gender, and microorganisms.*

References

1. Damiani, E., et al., *Effect of performance improvement programs on compliance with sepsis bundles and mortality: a systematic review and meta-analysis of observational studies.* PLoS One, 2015. **10**(5): p. e0125827.

For peer review only

2. Dellinger, R.P., et al., *Surviving sepsis campaign: international guidelines for management of severe sepsis and septic shock: 2012.* Crit Care Med, 2013. **41**(2): p. 580-637.
3. Gupta, R.G., et al., *Early goal-directed resuscitation of patients with septic shock: current evidence and future directions.* Crit Care, 2015. **19**: p. 286.
4. Rivers, E., et al., *Early goal-directed therapy in the treatment of severe sepsis and septic shock.* N Engl J Med, 2001. **345**(19): p. 1368-77.
5. Wisplinghoff, H., et al., *Nosocomial bloodstream infections in US hospitals: analysis of 24,179 cases from a prospective nationwide surveillance study.* Clin Infect Dis, 2004. **39**(3): p. 309-17.
6. Goto, M. and M.N. Al-Hasan, *Overall burden of bloodstream infection and nosocomial bloodstream infection in North America and Europe.* Clin Microbiol Infect, 2013. **19**(6): p. 501-9.
7. Holmbom, M., et al., *14-Year Survey in a Swedish County Reveals a Pronounced Increase in Bloodstream Infections (BSI). Comorbidity - An Independent Risk Factor for Both BSI and Mortality.* PLoS One, 2016. **11**(11): p. e0166527.
8. Mehl, A., et al., *Burden of bloodstream infection in an area of Mid-Norway 2002-2013: a prospective population-based observational study.* BMC Infect Dis, 2017. **17**(1): p. 205.
9. Skogberg, K., et al., *Population-based burden of bloodstream infections in Finland.* Clin Microbiol Infect, 2012. **18**(6): p. E170-6.
10. Sogaard, M., et al., *Temporal changes in the incidence and 30-day mortality associated with bacteremia in hospitalized patients from 1992 through 2006: a population-based cohort study.* Clin Infect Dis, 2011. **52**(1): p. 61-9.
11. Holmbom, M., et al., *Low incidence of antibiotic-resistant bacteria in south-east Sweden: An epidemiologic study on 9268 cases of bloodstream infection.* PLoS One, 2020. **15**(3): p. e0230501.
12. Laupland, K.B. and D.L. Church, *Population-based epidemiology and microbiology of community-onset bloodstream infections.* Clin Microbiol Rev, 2014. **27**(4): p. 647-64.
13. Ferrer, R., et al., *Empiric antibiotic treatment reduces mortality in severe sepsis and septic shock from the first hour: results from a guideline-based performance improvement program.* Crit Care Med, 2014. **42**(8): p. 1749-55.
14. Kumar, A., et al., *Initiation of inappropriate antimicrobial therapy results in a fivefold reduction of survival in human septic shock.* Chest, 2009. **136**(5): p. 1237-1248.
15. Liu, V.X., et al., *The Timing of Early Antibiotics and Hospital Mortality in Sepsis.* Am J Respir Crit Care Med, 2017. **196**(7): p. 856-863.
16. Vucelic, V., et al., *Sepsis and septic shock - an observational study of the incidence, management, and mortality predictors in a medical intensive care unit.* Croat Med J, 2020. **61**(5): p. 429-439.
17. Kumar, A., et al., *Duration of hypotension before initiation of effective antimicrobial therapy is the critical determinant of survival in human septic shock.* Crit Care Med, 2006. **34**(6): p. 1589-96.
18. Lane, D.J., et al., *Screening strategies to identify sepsis in the prehospital setting: a validation study.* CMAJ, 2020. **192**(10): p. E230-E239.
19. Park, J., et al., *Emergency medical service personnel need to improve knowledge and attitude regarding prehospital sepsis care.* Clin Exp Emerg Med, 2017. **4**(1): p. 48-55.
20. Sjosten, O., et al., *The prehospital assessment of patients with a final hospital diagnosis of sepsis: Results of an observational study.* Australas Emerg Care, 2019. **22**(3): p. 187-192.
21. Smyth, M.A., S.J. Brace-McDonnell, and G.D. Perkins, *Identification of adults with sepsis in the prehospital environment: a systematic review.* BMJ Open, 2016. **6**(8): p. e011218.
22. Rosenqvist, M., et al., *Improved Outcomes After Regional Implementation of Sepsis Alert: A Novel Triage Model.* Crit Care Med, 2020. **48**(4): p. 484-490.

For peer review only

23. Latten, G.H.P., et al., *Characteristics of the prehospital phase of adult emergency department patients with an infection: A prospective pilot study.* PLoS One, 2019. **14**(2): p. e0212181.
24. Seymour, C.W., et al., *Delays From First Medical Contact to Antibiotic Administration for Sepsis.* Crit Care Med, 2017. **45**(5): p. 759-765.
25. Widgren, B.R. and M. Jourak, *Medical Emergency Triage and Treatment System (METTS): a new protocol in primary triage and secondary priority decision in emergency medicine.* J Emerg Med, 2011. **40**(6): p. 623-8.
26. Widgren, B.R., M. Jourak, and A. Martinius, *[New accurate triage method. METTS-A yields basis for priority level decisions].* Lakartidningen, 2008. **105**(4): p. 201-4.
27. Vincent, J.L., et al., *The SOFA (Sepsis-related Organ Failure Assessment) score to describe organ dysfunction/failure. On behalf of the Working Group on Sepsis-Related Problems of the European Society of Intensive Care Medicine.* Intensive Care Med, 1996. **22**(7): p. 707-10.
28. *Royal College of Physicians. National Early Warning Score (NEWS) 2: Standardising the assessment of acute-illness severity in the NHS. Updated report of a working party. London: RCP, 2017.*
29. Ammerlaan, H.S., et al., *Secular trends in nosocomial bloodstream infections: antibiotic-resistant bacteria increase the total burden of infection.* Clin Infect Dis, 2013. **56**(6): p. 798-805.
30. Quan, H., et al., *Updating and validating the Charlson comorbidity index and score for risk adjustment in hospital discharge abstracts using data from 6 countries.* Am J Epidemiol, 2011. **173**(6): p. 676-82.
31. Quan, H., et al., *Coding algorithms for defining comorbidities in ICD-9-CM and ICD-10 administrative data.* Med Care, 2005. **43**(11): p. 1130-9.
32. Sundararajan, V., et al., *New ICD-10 version of the Charlson comorbidity index predicted in-hospital mortality.* J Clin Epidemiol, 2004. **57**(12): p. 1288-94.
33. Singer, M., et al., *The Third International Consensus Definitions for Sepsis and Septic Shock (Sepsis-3).* JAMA, 2016. **315**(8): p. 801-10.
34. *Empirical antibiotic treatment in severe infections.* 2021-08-26]; Available from: https://www.sls.se/globalassets/raf/ovriga-dokument/rodakortet-uppdaterat-150528.pdf.
35. *Swedish Strama antibiotic stewardship programme. Available; https://www.folkhalsomyndigheten.se/the-public-health-agency-of-sweden/communicable-disease-control/antibiotics-and-antimicrobial-resistance/swedish-work-on-containment-of-antibiotic-resistance/ Accessed 2021-03-10.*
36. *The European Committee on Antimicrobial Susceptibility Testing. Breakpoint tables for interpretation of MICs and zone diameters. Version 9.0, 2019. http://www.eucast.org.*
37. Andersson, M., et al., *Delay of appropriate antibiotic treatment is associated with high mortality in patients with community-onset sepsis in a Swedish setting.* Eur J Clin Microbiol Infect Dis, 2019.
38. Henriksen, D.P., et al., *Incidence rate of community-acquired sepsis among hospitalized acute medical patients-a population-based survey.* Crit Care Med, 2015. **43**(1): p. 13-21.
39. Jacobsson, G., E. Gustafsson, and R. Andersson, *Outcome for invasive Staphylococcus aureus infections.* Eur J Clin Microbiol Infect Dis, 2008. **27**(9): p. 839-48.
40. Melzer, M. and C. Welch, *Thirty-day mortality in UK patients with community-onset and hospital-acquired meticillin-susceptible Staphylococcus aureus bacteraemia.* J Hosp Infect, 2013. **84**(2): p. 143-50.
41. Whiles, B.B., A.S. Deis, and S.Q. Simpson, *Increased Time to Initial Antimicrobial Administration Is Associated With Progression to Septic Shock in Severe Sepsis Patients.* Crit Care Med, 2017. **45**(4): p. 623-629.
42. Seymour, C.W., et al., *Time to Treatment and Mortality during Mandated Emergency Care for Sepsis.* N Engl J Med, 2017. **376**(23): p. 2235-2244.

For peer review only

590 43. Lane, D., et al., *Prehospital management and identification of sepsis by emergency medical services: a systematic review.* Emerg Med J, 2016. **33**(6): p. 408-13.

592 44. Velasco, E., et al., *Bloodstream infection surveillance in a cancer centre: a prospective look at clinical microbiology aspects.* Clin Microbiol Infect, 2004. **10**(6): p. 542-9.

594 45. Corfield, A.R., et al., *Utility of a single early warning score in patients with sepsis in the emergency department.* Emerg Med J, 2014. **31**(6): p. 482-7.

596 46. Churpek, M.M., et al., *Quick Sepsis-related Organ Failure Assessment, Systemic Inflammatory Response Syndrome, and Early Warning Scores for Detecting Clinical Deterioration in Infected Patients outside the Intensive Care Unit.* Am J Respir Crit Care Med, 2017. **195**(7): p. 906-911.

600 47. Gerry, S., et al., *Early warning scores for detecting deterioration in adult hospital patients: systematic review and critical appraisal of methodology.* BMJ, 2020. **369**: p. m1501.

602 48. Rhodes, A., et al., *Surviving Sepsis Campaign: International Guidelines for Management of Sepsis and Septic Shock: 2016.* Intensive Care Med, 2017. **43**(3): p. 304-377.

For peer review only

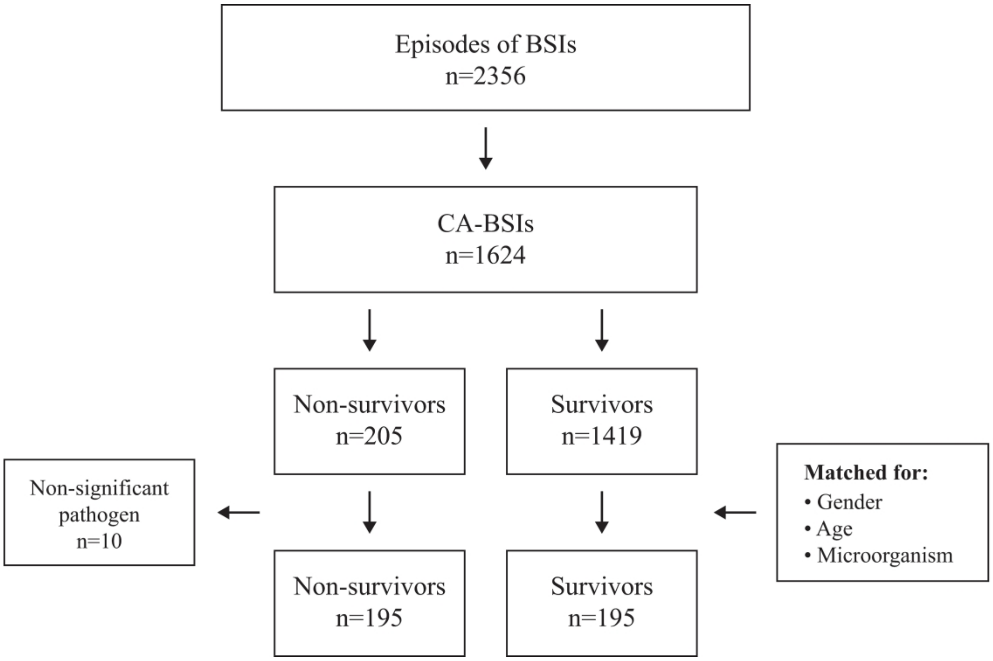

Figure 1. Flowchart of the study design

334x218mm (300 x 300 DPI)

Supplementary Table 1. Charlson Comorbidity Index

Comorbid Condition	ICD-9-CM Diagnosis Codes	ICD-10-CA Diagnosis Codes	Weight	Updated Weight
**Myocardial Infarction**	410, 412	I21, I22, I25.2	1	0
**Congestive Heart Failure**	398.91, 402.01, 402.11, 402.91, 404.01, 404.03, 404.11, 404.13, 404.91, 404.93, 425.4–425.9, 428 (hosp), 398, 402, 425, 428 (med)	I09.9, I11.0, I13.0, I13.2, I25.5, I42.0, I42.5–I42.9,I43, I50, P29.0	1	2
**Peripheral Vascular Disease**	093.0, 437.3, 440, 441, 443.1–443.9, 447.1, 557.1, 557.9, V43.3 (hosp) 440, 441, 443, 447, 557 (med)	I70, I71, I73.1, I73.8, I73.9, I77.1, I79.0, I79.2, K55.1, K55.8, K55.9, Z95.8, Z95.9	1	0
**Cerebrovascular Disease**	362.34, 430–438 (hosp) 430–438 (med)	G45, G46, H34.0, I60–I69	1	0
**Dementia**	290, 294.1, 331.2 (hosp) 290, 294, 331 (med)	F00–F03, F05.1, G30, G31.1	1	2
**Chronic Pulmonary Disease**	416.8, 416.9, 490–505, 506.4, 508.1, 508.8 (hosp), 416, 490–496, 500–505 (med)	I27.8, I27.9,J40–J47, J60–J67 J68.4, J70.1, J70.3	1	1
**Connective Tissue Disease-Rheumatic Disease**	446.5, 710.0–710.4, 714.0–714.2, 714.8, 725 (hosp), 446, 710, 714, 725 (med)	M05, M06, M31.5, M32–M34, M35.1, M35.3, M36.0	1	1
**Peptic Ulcer**	531–534	K25–K28	1	0
**Mild Liver Disease**	070.22, 070.23, 070.32, 070.33, 070.44, 070.54, 070.6, 070.9, 570, 571, 573.3, 573.4, 573.8, 573.9, V42.7 (hosp) 070, 570, 571, 573 (med)	B18, K70.0–K70.3, K70.9, K71.3–K71.5, K71.7, K73, K74, K76.0, K76.2–K76.4, K76.8, K76.9, Z94.4	1	2
**Diabetes without Chronic Complications**	250.0–250.3, 250.8, 250.9 (hosp) 250 (med)	E10.0, E10.1, E10.6, E10.8, E10.9, E11.0, E11.1, E11.6, E11.8, E11.9, E12.0, E12.1, E12.6, E12.8, E12.9, E13.0, E13.1, E13.6, E13.8, E13.9, E14.0, E14.1, E14.6, E14.8, E14.9	1	0
**Diabetes with Chronic Complications**	250.4–250.7 (med n/a)	E10.2–E10.5, E10.7, E11.2–E11.5, E11.7, E12.2–E12.5, E12.7, E13.2–E13.5, E13.7, E14.2–E14.5, E14.7	2	1
**Paraplegia or Hemiplegia**	334.1, 342, 343, 344.0–344.6, 344.9 (hosp), 334, 342–344 (med)	G04.1, G11.4, G80.1, G80.2, G81, G82, G83.0–G83.4, G83.9	2	2

For peer review only

**Renal Disease**	403.01, 403.11, 403.91, 404.02, 404.03, 404.12, 404.13, 404.92, 404.93, 582, 583.0–583.7, 585, 586, 588.0. V42.0, V45.1, V56 (hosp), 403, 582, 583, 585, 586, 588, V56 (med)	I12.0, I13.1, N03.2–N03.7, N052–N05.7, N18, N19, N25.0, Z49.0–Z49.2, Z94.0, Z99.2	2	1
**Cancer ***	140–172, 174–195.8, 200–208, 238.6 (hosp), 140–172, 174–195, 200–208, 238 (med)	C00–C26, C30–C34, C37–C41, C43, C45–C58, C60–C76, C81–C85, C88, C90–C97	2	2
**Moderate or Severe Liver Disease**	456.0–456.2, 572.2–572.4, 572.8 (hosp), 456, 572 (med)	I85.0, I85.9, I86.4, I98.2, K70.4, K71.1, K72.1, K72.9, K76.5–K76.7,	3	4
**Metastatic Carcinoma****	196–199	C77–C80	6	6
**HIV/AIDS**	042–044	B20–B22, B24	6	4

*Abbreviations: AIDS, acquired immunodeficiency syndrome; HIV, human immunodeficiency virus. * Including lymphoma and leukemia, except malignant neoplasm of skin ** or Metastatic solid tumour.*

For peer review only

Supplementary Table 2. Distribution of comorbidities

	Non-survivors n=195	Survivors n=195	p-value
**Comorbidity (%)**			
Myocardial infarction	31 (16)	41 (21)	0.19
Congestive heart failure	56 (29)	50 (26)	0.50
Peripheral vascular disease	14 (7)	16 (8)	0.70
Cerebrovascular disease	30 (15)	28 (14)	0.78
Dementia	15 (8)	11 (6)	0.42
Chronic pulmonary disease	35 (18)	33 (17)	0.79
Connective Tissue Disease-Rheumatic Disease	13 (7)	22 (11)	0.11
Peptic ulcer disease	3 (2)	3 (2)	>0.99
Mild liver disease	8 (4)	9 (5)	0.80
Diabetes without chronic complications	43 (22)	62 (32)	*0.03*
Diabetes with chronic complications	8 (4)	9 (5)	0.80
Paraplegia and hemiplegia	13 (7)	11 (6)	0.67
Renal disease	34 (17)	25 (13)	0.20
Cancer	67 (34)	44 (23)	*0.01*
Moderate or severe liver disease	9 (5)	3 (2)	0.08
Metastatic carcinoma	28 (14)	14 (7)	*0.02*
HIV/AIDS	0	0	-
Charlson (update weight) (SD)	3.2 (3.0)	2.3 (2.4)	*0.01*

Data are presented as no. (%) or mean (SD). Pearson chi2, Fisher's exact test or T-test, as appropriate.

For peer review only

Supplementary Table 3. Distribution of primary pathogen*

**Gram negative**	**Non-survivors n=195**	**Survivors n=195**	**%**
**Enterobacterales**			
Escherichia coli	41	41	21
Klebsiella pneumoniae	14	14	7
Klebsiella oxytoca	3	3	2
Enterobacter cloacae	2	2	1
Proteus Mirabilis	4	4	2
Haemophilus influenzae	4	4	2
**Other than Enterobacterales**			
Pseudomonas aeruginosa	7	7	4
**Others**	7	7	4
**Gram positives**			
**Enterococci**			
Enterococcus faecalis	6	6	3
Enterococcus faecium	5	5	3
**Staphylococci**			
Staphylococcus aureus	52	52	27
Staphylococcus spp	5	5	3
**Streptococci**			
Streptococcus pneumoniae	16	16	8
Streptococcus pyogenes (A)	1	1	1
Streptococcus agalactiae (B)	4	4	2
Streptococcus group C,G	5	5	3
Streptococcus spp	11	11	6
***Candida* spp**	4	4	2
**Others**	4	4	2
**Polymicrobial-BSI**	9	8	4

**Primary pathogen reported in the table. In Polymicrobial-BSI, an expert group (physicians specialised in infectious diseases) evaluated which pathogen was primary.*

For peer review only

Supplementary Table 4. Hospital data - Triage at hospital (RETTS and NEWS 2) and contact cause at hospital

	Non-survivors n=195	Survivors n=195	p-value
**Vital signs (SD)***			
Body temp (BT)	37.6 (1.3) n189	38.2 (1.1) n194	*<0.01*
Systolic blood pressure (SBP)	112 (28) n190	131 (28) n193	*<0.01*
Diastolic blood pressure (DBP)	65 (18) n188	72 (17) n192	*<0.01*
Pulse rate (HR)	102 (26) n190	97 (22) n192	*0.04*
Respiratory rate (RR)	27 (10) n184	23 (7) n189	*<0.01*
Blood O_2 saturation (SPO_2)	92 (9) n187	95 (4) n192	*<0.01*
PaO_2/FiO_2	42 (21) n183	51 (20) n187	*<0.01*
Reaction Level Scale (RLS)	1.4 (0.9) n187	1.1 (0.46) n192	*<0.01*
**Laboratory median (Q1-Q3)**			
C-reactive protein (mg/L)	180 (87-304) n195	138 (50-225) n192	*<0.01*
White blood cell count (x10^9/L)	13 (8-19) n195	13(10-17) n191	0.42
Platelet count (x10^9/L)	194 (130-292) n195	212 (156-280) n191	0.09
Creatinine (µmol/L)	143 (94-216) n191	98 (74-139) n190	*<0.01*
eGFR MDRD (mL/min/1.73 m^2)	36 (22-58) n186	58 (35-74) n186	*<0.01*
Albumin (g/L)	23 (20-28) n109	29 (25-32) n100	*<0.01*
B-Erythrocytes (x10^{12}/L)	4 (3-4) n195	4 (4-5) n191	*0.04*
B-MCV (fL)	95 (91-101) n195	93 (90-97) n191	*<0.01*
Haemoglobin (g/L)	116 (101-136) n195	121 (109-138) n191	*0.02*
Procalcitonin (µg/L)	10 (2-54) n34	4 (1-31) n26	0.33
**Primary focus of infection (%)**			
Respiratory tract	39 (20)	46 (24)	0.39
Urinary tract	42 (22)	50 (26)	0.34
Skin and soft tissue	19 (10)	20 (10)	0.86
Abdomen	12 (6)	18 (9)	0.25
CNS	-	2 (1)	0.50
Indwelling catheter	1 (1)	0	>0.99
Endocarditis	1 (1)	1 (1)	>0.99
Unknown	46 (24)	43 (22)	0.72
Missing data	35 (18)	15 (8)	
**Triage**			
RETTS Red (%)	76 (46) n164	62 (35) n177	*0.03*
RETTS Orange (%)	51 (31) n164	71 (40) n177	0.08
RETTS Yellow (%)	32 (20) n164	42 (24) n177	0.35
RETTS Green (%)	5 (3) n164	2 (1) n177	0.27
RETTS Red plus Orange (%)	127 (77) n164	133 (75) n177	0.62
NEWS 2 score (SD)	7.1 (4.0) n163	5.0 (3.4) n181	*<0.01*

For peer review only

NEWS 2 score ≥5 (%)	114 (70) n163	89 (49) n181	*<0.01*
**Reason for first hospital contact (%)**			
Fever	86 (44)	119 (61)	*<0.01*
Chills	26 (13)	50 (26)	*0.02*
"Found on the floor"	18 (9)	5 (3)	*0.01*
Gastrointestinal symptoms	32 (16)	32 (16)	>0.99
Cough	8 (4)	15 (8)	0.13
Dyspnoea/breathing difficulties	60 (31)	46 (24)	0.11
Urinary tract symptoms	13 (7)	25 (13)	*0.04*
Rapid deterioration in general condition	81 (42)	48 (25)	*<0.01*
Fatigue	14 (7)	17 (9)	0.57

*Data are presented as no. (%) or mean (SD). Pearson chi2, Fisher's exact test or T-test, as appropriate. Laboratory data are calculated with median, interquartile 25th to 75th percentile range (Q1-Q3) and Mann–Whitney U. * Vital signs first registered at admission. RETTS: Red = life-threatening condition, Orange = potentially life-threatening. Yellow/Green = acute process can wait.*

For peer review only

Supplementary Table 5. Early hospital care - Time for ambulance transport and time to empirical antibiotic treatment based on triage score (RETTS)

**Early hospital care**	**Non-survivors**	**Survivors**	**p-value**
Paramedic alarm times, hours, median (Q1-Q3)	0,72 (0,57-0,95), n137	0,73 (0,58-0,98), n131	0.40
Time to empirical antibiotic treatment based on triage score (RETTS), hours, median (Q1-Q3):			
Red (triage 1)	1,52 (0,67-3,18), n68	1,62 (0,90-2,98), n62	0.50
Orange (triage 2)	2,63 (1,72-5,15), n50	3,07 (1,63-6,13), n69	0.67
Yellow (triage 3)	4,88 (3,72-7,30), n32	5,28 (2,75-9,97), n38)	0.80
Green (triage 4)*	9,67 (6,26), n5	11,23 (8,60), n2	0.79

*Data are presented as hours and median (Q1-Q3) or * mean and SD. Mann-Whitney U test or T-test are used, as appropriate. RETTS: Red = life-threatening condition, Orange = potentially life-threatening. Yellow/Green = acute process can wait.*

For peer review only

Supplementary Table 6. Distribution of Empirical antibiotics used

	Non-survivors (n186)	Survivors (n192)	p-value
**Classes of antibiotics:**			
Aminoglycosides	15 (8)	9 (5)	0.18
Penicillins + beta-lactamase inhibitors	53 (28)	43 (22)	0.18
Carbapenems	23 (12)	8 (4)	<0.01
Cephalosporins	90 (48)	121 (63)	<0.01
Quinolones	3 (2)	1 (1)	0.37
Trimethoprim-sulfamethoxazole	0 (0)	1 (1)	>0.99
Glycopeptides	1 (1)	1 (1)	>0.99
Lincosamides	9 (5)	5 (3)	0.25
Macrolides	3 (2)	2 (1)	0.68
Nitroimidazoles	3 (2)	2 (1)	0.68
Penicillins	15 (8)	20 (10)	0.53
Antifungals	1 (1)	2 (1)	>0.99
Others	1 (1)	0 (0)	0.49
**Monotherapy treatment**	156 (84)	173 (90)	0.08
**Combination therapy treatment**	31 (17)	21 (11)	0.11

Data are presented as no. (%). Pearson chi2 or Fisher's exact test as appropriate. Combination therapy treatment was defined as more than one agent given within 4 hours in order to broaden the antimicrobial spectrum. Seven patients received oral antibiotic treatment, 4 non-survivors and 3 survivors.

For peer review only

STROBE Statement—Checklist of items that should be included in reports of case-control studies

	Item No	Page No
**Titel and abstract**	1	1
**Introduction**		
Background	2	3-4
Objectives	3	4
**Methods**		
Study design	4	4
Setting	5	4
Participants	6	4-5
Variables	7	4-7
Data sources	8	4-6
Bias	9	N/A
Study size	10	4-5
Quantitative variables	11	8
Statistical methods	12	8
**Results**		
Participants	13	9, Flow-chart
Descriptive data	14	9, table 1,2 and supplementary files
Outcome data	15	9-11, Table 1, 2
Main results	16	11-12
Other analyses	17	N/A
**Discussion**		
Key results	18	12
Limitations	19	15-16
Interpretation	20	12-15, 16
Generalisability	21	12-15, 16
**Other information**		
Funding	22	16

For peer review only

BMJ Open

Prehospital delay is an important risk factor for mortality in community-acquired bloodstream infection (CA-BSI): A matched case-control study

Journal:	*BMJ Open*
Manuscript ID	bmjopen-2021-052582.R2
Article Type:	Original research
Date Submitted by the Author:	30-Sep-2021
Complete List of Authors:	Holmbom, Martin; Linköping University, Department of Biomedical and Clinical Sciences; Linköping University, Department of Urology<br>Andersson, Maria; Linköping University, Division of Infectious Diseases, Department of Biomedical and Clinical Sciences, Faculty of Medicine and Health Sciences, Linköping University, Linköping, Sweden<br>Berg, Sören; Unit for Cardiovascular Sciences, Faculty of Medicine and Health Sciences<br>Eklund, Dan; Linköping University, Division of Infectious Diseases, Department of Biomedical and Clinical Sciences, Faculty of Medicine and Health Sciences, Linköping University, Linköping, Sweden<br>Sobczynski, Pernilla; Linköping University, Division of Infectious Diseases, Department of Biomedical and Clinical Sciences, Faculty of Medicine and Health Sciences, Linköping University, Linköping, Sweden<br>Wilhelms, Daniel; Linköping University, Department of Health and Medical Sciences,; Local Health Care Services in Central Östergötland, Department of Emergency Medicine<br>Moberg, Anna; Linköping University, Department of Health, Medicine and Caring Sciences, Linkoping University, Linkoping, Sweden<br>Fredrikson, Mats; Department of Clinical and Experimental Medicine, Faculty of Health Sciences<br>Balkhed, Åse Östholm; Linköping University, Division of Infectious Diseases, Department of Biomedical and Clinical Sciences, Faculty of Medicine and Health Sciences, Linköping University, Linköping, Sweden<br>Hanberger, Håkan ; Linköping University
**Primary Subject Heading**:	Infectious diseases
Secondary Subject Heading:	Emergency medicine, General practice / Family practice
Keywords:	Adult intensive & critical care < INTENSIVE & CRITICAL CARE, ACCIDENT & EMERGENCY MEDICINE, Public health < INFECTIOUS DISEASES, INFECTIOUS DISEASES, PRIMARY CARE

SCHOLARONE™
Manuscripts

I, the Submitting Author has the right to grant and does grant on behalf of all authors of the Work (as defined in the below author licence), an exclusive licence and/or a non-exclusive licence for contributions from authors who are: i) UK Crown employees; ii) where BMJ has agreed a CC-BY licence shall apply, and/or iii) in accordance with the terms applicable for US Federal Government officers or employees acting as part of their official duties; on a worldwide, perpetual, irrevocable, royalty-free basis to BMJ Publishing Group Ltd ("BMJ") its licensees and where the relevant Journal is co-owned by BMJ to the co-owners of the Journal, to publish the Work in this journal and any other BMJ products and to exploit all rights, as set out in our licence.

The Submitting Author accepts and understands that any supply made under these terms is made by BMJ to the Submitting Author unless you are acting as an employee on behalf of your employer or a postgraduate student of an affiliated institution which is paying any applicable article publishing charge ("APC") for Open Access articles. Where the Submitting Author wishes to make the Work available on an Open Access basis (and intends to pay the relevant APC), the terms of reuse of such Open Access shall be governed by a Creative Commons licence – details of these licences and which Creative Commons licence will apply to this Work are set out in our licence referred to above.

Other than as permitted in any relevant BMJ Author's Self Archiving Policies, I confirm this Work has not been accepted for publication elsewhere, is not being considered for publication elsewhere and does not duplicate material already published. I confirm all authors consent to publication of this Work and authorise the granting of this licence.

For peer review only

Prehospital delay is an important risk factor for mortality in community-acquired bloodstream infection (CA-BSI): A matched case-control study

Martin Holmbom[1,2], Maria Andersson[1], Sören Berg[3], Dan Eklund[1], Pernilla Sobczynski[1], Daniel Wilhelms[4], Anna Moberg[5], Mats Fredrikson[6], Åse Östholm Balkhed[1*], Håkan Hanberger[1*]

1 Division of Infectious Diseases, Department of Biomedical and Clinical Sciences, Faculty of Medicine and Health Sciences, Linköping University, Linköping, Sweden, **2** Department of Urology and Department of Biomedical and Clinical Sciences, Linköping University, Linköping, Sweden, **3** Unit for Cardiovascular Sciences, Faculty of Medicine and Health Sciences, **4** Department of Emergency Medicine in Linköping, and Department of Biomedical and Clinical Sciences, Linköping University, Linköping, Sweden **5** Department of Health, Medicine and Caring Sciences, Linkoping University, Linkoping, Sweden, **6** Department of Biomedical and Clinical Sciences and Forum Östergötland, Faculty of Medicine and Health Sciences. * Contributing equally to this work.

Corresponding author: Martin Holmbom, martin.holmbom@regionostergotland.se

For peer review only

ABSTRACT

Objectives: The aim of this study was to identify prehospital and early hospital risk factors associated with 30-day mortality in patients with blood culture-confirmed community-acquired bloodstream infection (CA-BSI) in Sweden.

Methods: A retrospective case-control study of 1,624 patients with CA-BSI (2015-2016), 195 non-survivors satisfying the inclusion criteria were matched 1:1 with 195 survivors for age, gender, and microorganism. All forms of contact with a healthcare provider for symptoms of infection within 7 days prior CA-BSI episode were registered. Logistic regression was used to analyse risk factors for 30-day all-cause mortality.

Results: Of the 390 patients, 61% (115 non-survivors and 121 survivors) sought prehospital contact. The median time from first prehospital contact till hospital admission was 13 hours (6-52) for non-survivors and 7 hours (3-24) for survivors (p<0.01). Several risk factors for 30-day all-cause mortality were identified: prehospital delay OR=1.26 (CI: 1.07-1.47), p<0.01; severity of illness (SOFA-score) OR=1.60 (CI: 1.40-1.83), p<0.01; comorbidity score (updated Charlson Index) OR=1.13 (CI: 1.05-1.22), p<0.01; and inadequate empirical antimicrobial therapy OR=3.92 (CI: 1.64-9.33), p<0.01. In a multivariable model, prehospital delay >24 hours from first contact

remained an important risk factor for 30-day all-cause mortality due to CA-BSI OR=6.17 (CI: 2.19-17.38), p<0.01.

Conclusion: Prehospital delay and inappropriate empirical antibiotic therapy were found to be important risk factors for 30-day all-cause mortality associated with CA-BSI. Increased awareness and earlier detection of bloodstream infection in prehospital and early hospital care is critical for rapid initiation of adequate management and antibiotic treatment.

Article summary

Strengths and limitations of this study

- Non-surviving patients were matched 1:1 with survivors for age, gender, and microorganism to evaluate risk factors for 30-day mortality.
- Data on limitation of level of care were collected and evaluated as a potential confounding factor.
- Only empirical antibiotic treatments were analysed, early changes in therapy were not considered.
- Data regarding patient delay *i.e.,* time from onset of symptoms to contact with prehospital or hospital care, were unavailable.

For peer review only

Background

Bloodstream infection (BSI) is a major cause of morbidity and mortality worldwide, with sepsis and septic shock as important complications [1-5]. In Europe, the number of BSIs and number of deaths due to BSI each year have been estimated to be 1.2 million and 157 000 respectively [6]. Most studies in Nordic countries report an increase in the incidence of BSI [7-10] and an increase in associated mortality [7, 9], but a low level of antibiotic resistance [11].

The increasing incidence of community-onset bloodstream infection has become a major problem. This may be due to an increase in healthcare-associated BSI related to complex medical care of aged patients with comorbidity in the community setting [7, 12]. BSI and the risk of transition to sepsis and septic shock with life-threatening organ dysfunction, requires early identification for prompt initiation of life-saving measures and improvement in outcome [13-17]. There are many studies focusing on improving early detection of patients at risk for developing sepsis in the Emergency Department (ED) [18-22]. Few studies have addressed prehospital care of sepsis prior to evaluation at the ED or by the paramedic [23, 24], and not one, to our knowledge, on the impact of delay in prehospital care on 30-day mortality in a well-defined case-control study on patients with community-acquired bloodstream infection (CA-BSI). The aim of this study was to identify prehospital and early hospital risk factors associated with 30-day mortality in patients with culture-confirmed CA-BSI

For peer review only

82 **METHODS**

83 **Study design and setting**

84 This was a case-control study on risk factors for 30-day mortality in patients with CA-BSI. From a
85 database of culture-confirmed CA-BSI in the Swedish county of Östergötland, samples of cases
86 (non-survivors) and matched controls (survivors) over a two-year period (from 1st January 2015
87 until 31st December 2016) were selected. The county of Östergötland, with a population of 445,000
88 in December 2015 and 452,000 in December 2016, had four hospitals, one tertiary care university
89 hospital, two general hospitals, one district hospital, 44 primary healthcare centres (PHCCs), and
90 four out-of-office hours centres.

91

92 In most West European countries, prehospital healthcare is available at several levels. In this study,
93 prehospital care is defined as healthcare contact prior to transport by ambulance or entering the
94 Emergency Department (ED). In Sweden, first contact with the healthcare service is usually by a
95 phone call to the PHCC or to the National Healthcare Guide (NHG). In urgent situations, the
96 emergency medical service (*i.e.*, paramedic ambulance service) is contacted directly. PHCCs
97 usually provide a call-back system where a nurse rings during office hours and assesses the need for
98 clinical assessment. The NHG offers immediate medical advice over the phone twenty-four hours a
99 day. It is manned by specially trained nurses using a decision-support algorithm, they assess the
100 need for further care and provide advice and/or recommend other healthcare services.

101

102 **Data collection**

103 From the microbiology laboratory database, the following dataset was collected: blood culture
104 results, number of aerobic and anaerobic blood culture vials taken, sites of puncture; species
105 identification, and susceptibility patterns. The dataset was entered into a second database where it

For peer review only

was linked to the patient-administration system providing the following data on all patients with a BSI episode: gender, age, comorbidity, admitting department, date of admission, date of discharge, and mortality.

A total of 2,356 BSI episodes were identified, of which 1,624 were CA-BSIs. To determine the impact of prehospital and early hospital care on 30-day all-cause mortality 2015-2016, we matched a group of non-survivors (cases) with survivors (controls) that satisfied the following inclusion criteria: adult (≥ 18 years), culture-confirmed CA-BSI with a significant pathogen, registered in the County of Östergötland, treated in a hospital in the County of Östergötland, non-survivor (dead within 30 days from positive blood culture) and survivor alive at (30 days).

In all, 195 non-survivors met the inclusion criteria and were matched with 195 survivors for age (+/- 10 years), gender, and microorganisms (Fig 1).

For non-survivors and survivors data on all infection-related contacts with a healthcare provider (telephone contact or physical contact) ≤7 days prior to a positive blood culture were collected by reviewing medical records. For each contact, the following were documented in the Case Report Form: profession of healthcare provider, suspected diagnosis, vital signs, primary focus of infection, and initial advice given. In the event of ambulance transport, information on time to reach the patient, time to reach ED, vital signs, intravenous fluid administration, and oxygen administration were extracted from the mission report.

The following data on management in the ED were collected: limitation of level of care, medical personnel involved, vital signs, laboratory data, consultation with a specialist if any (infectious diseases, internal medicine, anaesthesia *etc.*), time on the ED, times to fluid administration and antibiotic treatment, other sepsis treatment such as corticosteroids, and highest serum lactate within 24 hours after admission. The highest priority given by the triage system RETTS (Rapid Emergency

For peer review only

Triage and Treatment System) was documented [25, 26]. Sequential Organ Failure Assessment (SOFA) score [27] and National Early Warning Score (NEWS) 2 [28] were also calculated.

An expert group consisting of physicians specialised in infectious diseases judged the correctness of the antibiotic treatment based on local recommendations for empirical treatment of the suspected focus at that time, and the pathogen finally cultured. Doses of antibiotics, corrected for renal function, were also judged. Comorbidity scores according to the modified Charlson Comorbidity Index, based on diagnoses obtained from patient medical records up to 24 months prior to the BSI, were later entered into the database.

Definitions

Blood culture: One set of blood cultures comprised one aerobic and one anaerobic blood culture bottle. It was recommended that at least two sets of blood cultures be taken simultaneously.

Positive blood culture: The isolations of microorganism/s (one or more bacterial or fungal isolates) from a set of blood cultures. Only bacterial or yeast isolates from initial blood cultures were considered; repeat isolates were excluded.

Non-significant pathogens: Microorganisms typically belonging to the skin microbiome (Coagulase-Negative Staphylococci, (CoNS), *Micrococcus* spp., *Bacillus* spp., *Corynebacterium* spp. and *Propionibacterium* spp.) were considered probable contaminants and excluded [29]. An exception was CoNS isolated from blood cultures taken from at least two different puncture sites on the same occasion, and with an identical resistance pattern.

Community-acquired bloodstream infection (CA-BSI): A positive blood culture obtained within 48 hours after hospital admission. Cultures from readmission within 30 days were excluded. Furthermore, patients who had received intravenous treatment or advanced nursing care at home such as parenteral nutrition, haemodialysis, or intravenous chemotherapy, within the previous 30 days were excluded.

For peer review only

Immunosupression: Patients were deemed immunocompromised if prescribed immunosuppressive medication or had a code number (D80-D89) for an immunosuppressive disease according to the International Classification of Diseases, 10th edition (ICD-10) when admitted to the hospital.

Comorbidity: Comorbidity score was based on the updated Charlson Comorbidity Index [30-32] using ICD-10 diagnosis codes documented in the patient's medical record up to 24 months prior to date of admission (Supplementary File, Table 1).

Sepsis: The Sepsis-3 definition [33] was used *i.e.*, an increase by 2 points or more in the SOFA score due to infection.

Limitation of level of care: In some cases, the decision was made to limit the level of care. Such a decision influences treatment provided and thus mortality. This is an obvious confounding factor. Limitation of level of care set in the ED or within the first 24 h of admission was registered as: (a) withholding of intensive care (no ICU); (b) withholding of cardiopulmonary resuscitation (no CPR); and (c) withdrawal of all active treatment *i.e.*, end-of-life care (EoLC).

Appropriate empirical antibiotic therapy: Defined by local recommendations, suspected source of infection, severity of illness, dosage, and correction for renal function [34, 35].

Microbiologically appropriate empirical antibiotic therapy: Defined according to cultured pathogen and its susceptibility patterns [34-36].

For peer review only

Microbiological methods

All microorganisms isolated were analysed at the species level. Microorganism identification and susceptibility testing were performed at the Clinical Microbiology Department, University Hospital, Linköping using matrix-assisted laser desorption ionisation time-of-flight mass spectrometry (MALDI-TOF MS). Antibiotic susceptibility classification used during study period was: Susceptible (S), Intermediate (I), and Resistant (R), according to the European Committee on Antimicrobial Susceptibility Testing (EUCAST) guidelines [36].

Statistical analysis

Data are presented as mean and standard deviation (SD) or median and quartiles (Q1-Q3). We compared normally distributed quantitative variables using the Student *t* test and categorical variables using the chi^2 test or Fisher exact test as appropriate. Mann–Whitney U test and Kruskal Wallis test were used otherwise. A two-tailed p value < 0.05 was considered statistically significant. Correlations were analysed according to Pearson. Missing data were treated by pairwise deletion, and there was no imputation. Univariate and multivariable analyses were performed with logistic regression including the descriptive parameters that correlated to 30-day mortality. Two patients among the non-survivors had EoLC taken prior to admission to the hospital. This was considered to have had a major effect on early treatment and time to admission to hospital for these patients and they and their matched survivors were excluded from the analyses regarding time to admission and the multiple regression model. The statistics programme SPSS version 25 was used for the analyses.

For peer review only

Results

Demographic and clinical characteristics

A total of 2,356 BSI episodes were identified during the study period, of which 1,624 (69%) were CA-BSIs. The all-cause 30-day mortality rate of CA-BSI was 12.6% (n=205). Of these, 95% (n=195) met the inclusion criteria and were included in the study as non-survivors (case-group) and were matched with 195 survivors (control group) for gender, age, and microorganism. Almost 40% of the non-survivors died within 3 days after admission, and 80% within 7 days. Underlying comorbidity scores according to the updated Charlson Comorbidity Index were higher among non-survivors than survivors (3.2 vs 2.3, p=<0.01). Non-survivors were more often diagnosed with cancer (34% vs 23%, p=0.01) and metastatic carcinoma (14% vs 7%, p=0.02) compared to survivors. Limitation of level of care before admission was significantly more common among non-survivors (14% vs 2%, p=<0.01), (Table 1 and Supplementary File, Table 2).

Microbiology

There were 407 isolates from 390 cases. *Staphylococcus aureus* was the most frequently isolated primary pathogen (27%), followed by *Escherichia coli* (21%), *Streptococcus pneumoniae* (8%) and *Klebsiella pneumoniae* (7%). *Candida* spp. were observed in 2% of cases. Polymicrobial CA-BSI was found in 9 non-survivors and 8 survivors. Microbiological findings are summarised in Supplementary File, Table 3.

For peer review only

Table 1. Demographics and prehospital data

	Non-survivors n=195	Survivors n=195	p-value
**Demographics (%)**			
Male	106 (54)	106 (54)	>0.99
Mean age (SD)	78y (±13)	76y (±12)	0.20
Surgery within 30d	29 (15)	27 (14)	0.77
Immunosuppression	60 (31)	45 (23)	0.09
**Charlson (update weight) (SD)**	3.2 (2.99)	2.3 (2.36)	*0.01*
**Patients with any limitation of level of care before admission (%)**	28 (14)	4 (2)	*<0.01*
EoLC	2 (1)	0	0.50
No ICU	17 (9)	2 (1)	*<0.01*
No CPR	27 (14)	4 (2)	*<0.01*
**First prehospital contact site (%)**			
Phone to NHG	14 (7)	60 (31)	*<0.01*
Phone to a PHCC	79 (41)	33 (17)	*<0.01*
Patient visit to a PHCC	22 (11)	28 (14)	0.36
No prehospital contact	80 (41)	74 (38)	0.53
**Multiple prehospital contacts (≥2) (%)**	64 (33)	41 (21)	*0.01*
**Reason for first prehospital contact (number) (%)**	n115	n121	
Fever	44 (38)	43 (36)	0.67
Chills	13 (11)	13 (11)	0.89
"Found on the floor"	4 (3)	0	*0.04*
Gastrointestinal symptoms	16 (14)	23 (19)	0.29
Cough	3 (3)	13 (11)	*0.01*
Dyspnoea/breathing difficulties	23 (20)	15 (12)	0.11
Urinary tract symptoms	14 (12)	22 (18)	0.20
Rapid deterioration in general condition	47 (41)	29 (24)	*<0.01*
Fatigue	3 (3)	15 (12)	*<0.01*
**Time from first prehospital contact to admission, hours, median (Q1-Q3)**	13 (5.9-51.6) n113	7.2 (3.3-24) n119	*<0.01*
Phone to NHG	4 (1.0-8.7) n13	3.6 (1.3-7.3) n58	0.64
Phone to a PHCC	24 (6.8-72) n78	12.5 (5.9-24) n33	0.06
Visit to a PHCC	11 (6.6-77) n22	12.5 (6.4-66) n28	0.75
**Time from first prehospital contact to admission, intervals (%)**	n193	n193	
0-6 hours	29 (15)	49 (25)	*0.01*
6-12 hours	26 (14)	30 (16)	0.56
12-24 hours	12 (6)	21 (11)	0.10
>24 hours	46 (24)	19 (10)	*<0.01*
No prehospital contact	80 (41)	74 (38)	0.53

Data are presented as no. (%) or mean (SD). Pearson chi2, Fisher's exact test or T-test, as appropriate. P values < 0.05 are shown in italics. Time indications are calculated with median, interquartile 25th to 75th percentile range (Q1-Q3) and Mann–Whitney U. Reason for first prehospital contact: more than one reason possible. NHG = national healthcare guide. PHCC=primary healthcare centre.

For peer review only

Prehospital data

Of the 390 patients, 61% (115 non-survivors and 121 survivors) had taken contact with a prehospital healthcare facility for infection-related symptoms within 7 days prior to admission to hospital. Forty-four per cent of these patients had ≥2 prehospital contacts. Forms of prehospital contact were phone contact with a PHCC (47%), phone contact with NHG (31%), and visit to a PHCC (21%). Fever was the primary reason for prehospital contact (37%). Rapid deterioration in general condition was the second most common reason (32%) and was more common among non-survivors than survivors (41% vs 24%, p<0.02) (Table 1). Phone contact with a PHCC was the more common form of first contact among non-survivors (41% non-survivors vs 17% survivors, p=0.01), whereas phone contact with NHG was more common among survivors (7% non-survivors vs 31% survivors, p=<0.01) (Table 1).

Time from first infection-related prehospital contact till hospital admission

In total, the median time from first prehospital contact till hospital admission was 8 hours (4-27). This was significantly longer for non-survivors 13 hours (6-52) vs 7.2 hours (3-24) for survivors, p=0.01 (Table 1). For the study group as a whole, there were significant differences in time to admission depending on form of first contact, with shorter median time to admission after telephone contact with the NHG 3.8 hours (1.3-7.7) compared to phone contact with a PHCC 24.0 hours (6.8-48) or visit to a PHCC 11.3 hours (6.7-72) (p<0.01).

For peer review only

Hospital data

A total of 88% were admitted to hospital via the ED (85% non-survivors vs 91% survivors, p=0.04), other patients were admitted via a specialist outpatient department. Hospital admission after ambulance transport was more common among non-survivors (81% vs 70%, p=0.01) (Table 2). The most common sign reported when admitted to hospital was fever (54%). The most common infection focus was a urinary tract infection (24%) followed by infection with unknown focus (23%), and respiratory tract infection (22%). There were no differences between non-survivors and survivors regarding primary focus of infection (Supplementary File, Table 4).

On the ED, vital signs and most laboratory test results indicated a more serious condition in non-survivors compared to survivors and 159 non-survivors and 161 survivors were triaged with a life-threatening or potentially life-threatening condition according to the RETTS score. Abnormalities in respiratory function, haemodynamics, and neurological function were significantly more prominent among non-survivors (Supplementary File, Table 4). SOFA score on admission and at 24 hours was higher among non-survivors 4.2 (SD: 2.3) vs 2.3 (SD: 1.7), (p<0.01) and 6.8 (3.6) vs 3.8 (2.7) (p<0.01) respectively). On admission to hospital, 75% of non-survivors and 52% of survivors (p<0.01) fulfilled the Sepsis 3 criteria, and within 24 hours the corresponding figures were 95% of non-survivors and 79% of survivors (p<0.01). In all, 11% of non-survivors received ICU care within 24 hours compared to 5% of survivors (p=0.01) (Table 2).

For peer review only

Table 2. Hospital data - Severity of diseases and antibiotic treatment

	Non-survivors n=195	Survivors n=195	p-value
**Ambulance transport (%)**	158 (81) n194	136 (70) n193	*0.01*
**Admission to hospital (%)**			
Emergency department	165 (85)	178 (91)	*0.04*
Specialist outpatient department	30 (15)	17 (9)	*0.04*
**Severity of disease (SD)**			
Habitual SOFA	0.8 (1.1) n185	0.4 (0.8) n193	*<0.01*
SOFA score on admission	4.2 (2.3) n161	2.3 (1.7) n160	*<0.01*
SOFA score at 24 h	6.8 (3.6) n 187	3.8 (2.7) n 195	*<0.01*
Maximum lactate level mmol/l first 24 hr	4.6 (4.0) n 122	2.9 (1.9) n 110	*<0.01*
NEWS 2	7.1 (4.0) n 163	5.0 (3.4) n 181	*<0.01*
**Sepsis (%)**			
Sepsis on admission	119 (75) n158	82 (52) n158	*<0.01*
Sepsis at 24 h	176 (95) n185	152 (79) n193	*<0.01*
**ICU care within 24 hours (%)**	22 (11.3)	9 (4.6)	*0.01*
**Antibiotic treatment**			
Antibiotic administration (%)	186 (96) n193	192 (99.5) n193	0.07
Time (hours) to antibiotics from admission median, (Q1-Q3)	2.8 (1.4-5.3) n185	3.0 (1.4-6.2) n192	0.58
First dose antibiotic within one hour (%)	34 (18) n185	30 (16) n192	0.48
Appropriate empirical antibiotic therapy (%)	162 (87) n186	185 (96) n192	*<0.01*
Microbiologically appropriate empirical antibiotic therapy (%)	155 (83) n186	168 (88) n192	0.25
**Intravenous fluids in the ED (%)**	124 (76) n163	116 (68) n171	0.09

Data are presented as no. (%) or mean (SD). Pearson chi2, Fisher's exact test or T-test, as appropriate. P values < 0.05 are shown in italics. Time indications are calculated with median, interquartile 25th to 75th percentile range (Q1-Q3) and Mann–Whitney. Two non-survivors with end-of-life care (EoLC) on arrival at hospital, and their controls were excluded from the analysis regarding antibiotic treatment and intravenous fluids

Antibiotic treatment was initiated in 96% of non-survivors and 99.5% of survivors, (p=0.07). The median time from hospital admission to start of empirical antibiotic did not differ between the groups, non-survivors 2.8 hours (1.4-5.3) and survivors 3.0 hours (1.4-6.2), nor in the case of sub-classification according to triage score (RETTS) (Supplementary File, Table 5). Antibiotic administration was started within one hour in 18% of non-survivors and 16% of survivors (p=0.48). More survivors received appropriate empirical antibiotic therapy than non-survivors (87% non-survivors and 96% survivors, p<0.01). Microbiologically appropriate empirical antibiotic therapy did not differ significantly between the groups (82% non-survivors vs 88% survivors) (Table 2). The empirical antibiotics used are listed in Supplementary File, Table 6.

For peer review only

Analysis of risk factors for 30-day mortality in CA-BSI

In the univariate logistic regression several risk factors associated with 30-day mortality were observed: comorbidity score (updated Charlson Index) OR = 1.13 (CI 1.05-1.22), p<0.01, prehospital delay OR = 1.26 (CI 1.07-1.47), p=0.01, severity of illness (SOFA score on admission) OR = 1.60 (CI 1.40-1.83), p<0.01 and inappropriate empirical antibiotic therapy OR = 3.92 (CI 1.64-9.33), p<0.01 (Table 3).

In the multivariable model of 30-day mortality, prehospital delay >24 hours was significantly related to mortality, OR = 6.17 (CI 2.19-17.38), p<0.01. This was followed by inappropriate empirical antibiotic therapy, OR = 5.50 (CI 1.62-18.63), p=0.01, no prehospital contact, OR = 2.56 (CI 1.02-6.41), p=0.045, and severity of illness (SOFA score on admission), OR = 1.35 (CI 1.16-1.57), p<0.01 (Table 3).

Table 3. Risk factors for 30-day mortality

	**Univariate analysis**			**Multivariable analysis**		
**Risk factor**	**OR**	**95% CI**	**p-value**	**OR***	**95% CI**	**p-value**
**Time from first prehospital contact to admission (intervals)**	1.26	1.07-1.47	*0.01*			
0-6h	1**			1**		
6-12h	1.46	0.73-2.94	0.28	1.35	0.48-3.79	0.56
12-24h	0.97	0.42-2.25	0.94	0.56	0.15-2.10	0.39
>24h	4.09	2.02-8.28	*<0.01*	6.17	2.19-17.38	*<0.01*
No prehospital contact	1.83	1.05-3.19	*0.03*	2.56	1.02-6.41	0.05
**Updated Charlson**	1.13	1.05-1.22	*<0.01*	1.02	0.91-1.15	0.70
**Ambulance transport**	1.84	1.14-2.97	*0.01*	1.59	0.68-3.71	0.29
**Admission through ED**	0.55	0.29-1.03	0.06	1.35	0.43-4.25	0.61
**In SOFA score**	1.60	1.40-1.83	*<0.01*	1.35	1.16-1.57	*<0.01*
**Inappropriate empirical antibiotic therapy***	3.92	1.64-9.33	*<0.01*	5.50	1.62-18.63	*0.01*
**Rapid deterioration in general condition**	1.79	1.06-3.02	*0.03*	2.27	0.98-5.28	0.06
**Any care restrictions before or within 24h after admission**	9.95	5.95-16.64	*<0.01*	10.37	4.96-21.67	*<0.01*
**Matching**	1	0.99-1.00	>0.99	1	0.99-1.0	0.20

** Multivariable binomial regression analysis. ** Reference. *** Clinical inappropriateness defined by local recommendations, suspected source of infection, severity of illness, dosage, and correction for renal function.*

For peer review only

Discussion

This study revealed that prehospital delay had a major impact on mortality among patients with CA-BSI. Non-survivors were more seriously ill when admitted to hospital, with significantly higher SOFA scores and more signs of sepsis. Although initial care at the hospital and time to antibiotic treatment were comparable between non-survivors and survivors (for some variables even better among non-survivors) this was not enough to compensate for the negative effect of prehospital delay.

CA-BSI patients form a heterogeneous group in which underlying disease, gender, age, and appropriate management vary significantly. The severity of bloodstream infection ranges from asymptomatic bacteraemia to fulminant sepsis or septic shock. To our knowledge, this is the first study to focus on a well-defined population of adult patients with CA-BSI focusing on prehospital care prior to hospital admission. In previous study by Holmbom *et al*, gender, age, and pathogen were risk factors for mortality in BSI [7, 11], so these were used when matching patients to enable evaluation of other potential risk factors in this study. Compared to previous studies and reports on sepsis and CA-BSI, the distribution of pathogens in the present study was different [7, 37, 38], with *S.aureus* being the most common. This was probably a result of the study design *i.e.,* inclusion of patients dying within 30 days and matched controls, since *S.aureus* BSI has such a high mortality rate [39, 40].

Delay in antibiotic treatment has previously been shown to have a clear association with increased mortality in progressive sepsis and septic shock [13, 15, 17, 37, 41, 42]. However, delay prior to ambulance transport or coming to the ED, and causes of delay have hardly been investigated [24]. Strategies for early identification of sepsis in prehospital care, and measures to increase survival from sepsis have received much attention, and several strategies and screening tools have been investigated with varied results [18, 21, 43]. Among the patients in the present study who took prehospital contact with the healthcare service, form of first prehospital contact affected prehospital

For peer review only

delay. Telephone contact with the NHG was associated with shorter time to hospital admission than phone contact or visit to the local PHCC. The reason for this remains unclear. Possible factors include: 1. Compliance to the NHG computer-based decision algorithm designed for acute illness, as well as nurses more experienced in giving advice over the phone; 2. More liberal attitude of NHG nurses to send patients to the ED; 3. Non-survivors have higher comorbidity scores and tend to seek initial contact with their PHCC. Frail patients have also greater difficulty in describing their symptoms, making it more difficult for the primary healthcare provider to evaluate the seriousness of their condition; and 4. Whereas the NHG gives advice directly, it is possible for the PHCC to delay clinical assessment until later the same or next day. In fact, the call-you-back system used by PHCCs may have caused even more delay than seen in this study since we did not have access to when the first phone call from the patient was made. Instead, we used the time the phone-call back to the patient was made. In conclusion, the BSI healthcare process and its outcome varies according to the form of healthcare provider the patient first contacts.

We also looked for factors facilitating earlier identification of patients with increased risk for death. The comorbidity score (updated Charlson Comorbidity Index) was higher among non-survivors, with higher rates of cancer and metastatic carcinoma. These conditions are associated not only with increased mortality risk in general but also with BSI itself [7, 11, 44]. In prehospital records, “found on the floor” and “rapid deterioration in general condition” were more often reported among non-survivors. Since both reflect a seriously ill person, the patient is by proxy identified as a patient at risk. Although early prehospital identification of patients at high risk for sepsis or mortality is complex [43], we confirm the findings of previous studies [7, 11, 44] that taking signs and symptoms of infection in cancer patients seriously is crucial. Otherwise, we found no patient-specific factors that could be of help in identifying patients with increased mortality risk.

For peer review only

Many studies on sepsis, especially prior to Sepsis 3, have had patients with a wide spectrum of disease severity. This has certainly contributed to differences in results regarding the impact of time to antibiotic treatment on outcome [13-15, 17]. Our study comprised patients with CA-BSI, not true sepsis, and thus even more likely to be heterogeneous. Even so, 75% of non-survivors and 52% of survivors had sepsis on admission to the ED. The severity of illness on admission to hospital was greater in non-survivors and there are several factors that might explain this. One factor could be a longer prehospital delay as observed in this study.

Previous studies have shown the importance of rapid detection and initiation of adequate antibiotic treatment in sepsis and especially septic shock [13-15, 17, 37]. Nevertheless, using the RETTS triage system [25, 33], only half of the non-survivors were captured by the highest priority (red). Furthermore, priorities red plus orange together would still have failed to detect one third. Likewise, when using NEWS 2 with the proposed cut-off score of 5 [28, 45], at least one third of the non-survivors would have remained undetected as patients at risk for sepsis in the initial triage. Triage is useful when escalating the care of patients already identified with sepsis, as shown by Rosenqvist *et al* [22], but we lack a valid, simple, highly predictive scoring system to detect sepsis [46, 47]. Surviving Sepsis Guidelines 2016 recommend that antibiotics be administered within one hour of diagnosing sepsis [48]. This was not achieved in our material where only 16% of non-survivors and 18% of survivors received the first dose of antibiotics within one hour. Appropriate empirical antibiotic therapy according to local guidelines was more common among survivors ($p<0.01$), while microbiologically appropriate empirical antibiotic therapy was not. This could indicate that not only correct empirical antibiotics is important, but that also a holistic view of the patient, including suspected focus of infection and need of source control, are important parts of early management. This study showed that, prehospital delay and non-appropriate empirical treatment among patients with CA-BSI worsens the outcome and measures should be taken both pre-hospital and at the emergency unit to increase survival in CA-BSI.

For peer review only

Limitations

To our knowledge, this is the first study on a well-defined population of adult patients with CA-BSI, focusing on prehospital care prior to hospital admission, but it has limitations.

1. Though non-survivors and survivors were matched for gender, age (+/- 10 years), and microorganism, microorganism resistance patterns were not considered when matching non-survivors and survivors. However, since the prevalence of resistant bacteria in Sweden is low, this could only have had a minor influence on the results. Furthermore, when analysing correct antimicrobial therapy based on microorganisms cultured and their resistance patterns, there was no significant difference between the groups.
2. We only analysed the correctness of empirical antibiotic treatment, while early changes in therapy were not considered.
3. Other causes of death such as myocardial infarction, respiratory failure or pulmonary embolism as primary cause of death were possible, but such conditions would likely be related to the underlying infection, and since 95% of the cases had a fulminant sepsis within 24 hours this would be the major predisposing factor.
4. The only differences in prehospital management affecting outcome in this study were the timing of events. Other variables require further research.
5. The only time parameters for early hospital care in this study were time to empirical antibiotic treatment and time for ambulance transport. Presumably, time to antibiotic treatment also implies time to early hospital treatment. Furthermore, time to early hospital care is usually based on severity of illness (RETTS and NEWS 2) when admitted to hospital. Time to antibiotic treatment based on RETTS did not differ between the groups. This study was not designed to validate the time to care based on triage level, RETTS or NEWS 2 correctness.

For peer review only

6. Community healthcare in Sweden has several healthcare providers i.e., care provided by the community, private medicine and/or self-medication (by patient or relative). This result in unavailable data, and it is possible that some community-acquired healthcare-associated BSIs may have occurred in the study population, even though hospital and healthcare-related infections were excluded as far as possible.
7. Data regarding patient delay *i.e.,* time from onset of symptoms to contact with prehospital or hospital care should be included when calculating hospital and therapy delay. However, these were unavailable since patient delay was not systematically registered in the patient records.

For peer review only

Conclusion

In this case-control study, prehospital delay and inappropriate empirical antibiotic therapy were found to be important risk factors for 30-day all-cause mortality associated with CA-BSI. Effective guidelines to aid recognition of patients likely to develop sepsis in prehospital and early hospital care, and an effective prehospital medical advice service, could help to make significant progress in the care of patients with bloodstream infection.

Acknowledgements

Thanks to consultant anaesthetist Peter Cox for proof reading.

Competing interests

None declared.

Author Contributions

MH, HH and ÅÖB conceived and designed the study. MH, MA, DE, ÅÖB and MF analysed the data. MH and MA wrote the manuscript. All authors (MH, MA, SB, DE, PS, DW, AM, MF, ÅÖB

and HH) contributed to the discussion and reviewed the manuscript. All authors (MH, MA, SB, DE, PS, DW, AM, MF, ÅÖB and HH) commented and approved the final version of the paper.

Data Availability

All relevant data are within the manuscript and its Supplementary File.

Funding

This work was supported by Östergötland Count Council (Award/grant number: N/A).

Patient and public involvement

There was no patient and/or public involvement in the design of the study.

Ethics approval

The Regional Ethics Review Board in Linköping, Sweden, approved the study (Ref. no: 2010/160-31).

Figure legends

Fig 1. *A total of 2,356 BSI episodes were identified, of which 1,624 were CA-BSIs, 195 non-survivors met the inclusion criteria and were matched with 195 survivors for age (+/- 10 years), gender, and microorganisms.*

References

1. Damiani, E., et al., *Effect of performance improvement programs on compliance with sepsis bundles and mortality: a systematic review and meta-analysis of observational studies.* PLoS One, 2015. **10**(5): p. e0125827.

For peer review only

2. Dellinger, R.P., et al., *Surviving sepsis campaign: international guidelines for management of severe sepsis and septic shock: 2012.* Crit Care Med, 2013. **41**(2): p. 580-637.
3. Gupta, R.G., et al., *Early goal-directed resuscitation of patients with septic shock: current evidence and future directions.* Crit Care, 2015. **19**: p. 286.
4. Rivers, E., et al., *Early goal-directed therapy in the treatment of severe sepsis and septic shock.* N Engl J Med, 2001. **345**(19): p. 1368-77.
5. Wisplinghoff, H., et al., *Nosocomial bloodstream infections in US hospitals: analysis of 24,179 cases from a prospective nationwide surveillance study.* Clin Infect Dis, 2004. **39**(3): p. 309-17.
6. Goto, M. and M.N. Al-Hasan, *Overall burden of bloodstream infection and nosocomial bloodstream infection in North America and Europe.* Clin Microbiol Infect, 2013. **19**(6): p. 501-9.
7. Holmbom, M., et al., *14-Year Survey in a Swedish County Reveals a Pronounced Increase in Bloodstream Infections (BSI). Comorbidity - An Independent Risk Factor for Both BSI and Mortality.* PLoS One, 2016. **11**(11): p. e0166527.
8. Mehl, A., et al., *Burden of bloodstream infection in an area of Mid-Norway 2002-2013: a prospective population-based observational study.* BMC Infect Dis, 2017. **17**(1): p. 205.
9. Skogberg, K., et al., *Population-based burden of bloodstream infections in Finland.* Clin Microbiol Infect, 2012. **18**(6): p. E170-6.
10. Sogaard, M., et al., *Temporal changes in the incidence and 30-day mortality associated with bacteremia in hospitalized patients from 1992 through 2006: a population-based cohort study.* Clin Infect Dis, 2011. **52**(1): p. 61-9.
11. Holmbom, M., et al., *Low incidence of antibiotic-resistant bacteria in south-east Sweden: An epidemiologic study on 9268 cases of bloodstream infection.* PLoS One, 2020. **15**(3): p. e0230501.
12. Laupland, K.B. and D.L. Church, *Population-based epidemiology and microbiology of community-onset bloodstream infections.* Clin Microbiol Rev, 2014. **27**(4): p. 647-64.
13. Ferrer, R., et al., *Empiric antibiotic treatment reduces mortality in severe sepsis and septic shock from the first hour: results from a guideline-based performance improvement program.* Crit Care Med, 2014. **42**(8): p. 1749-55.
14. Kumar, A., et al., *Initiation of inappropriate antimicrobial therapy results in a fivefold reduction of survival in human septic shock.* Chest, 2009. **136**(5): p. 1237-1248.
15. Liu, V.X., et al., *The Timing of Early Antibiotics and Hospital Mortality in Sepsis.* Am J Respir Crit Care Med, 2017. **196**(7): p. 856-863.
16. Vucelic, V., et al., *Sepsis and septic shock - an observational study of the incidence, management, and mortality predictors in a medical intensive care unit.* Croat Med J, 2020. **61**(5): p. 429-439.
17. Kumar, A., et al., *Duration of hypotension before initiation of effective antimicrobial therapy is the critical determinant of survival in human septic shock.* Crit Care Med, 2006. **34**(6): p. 1589-96.
18. Lane, D.J., et al., *Screening strategies to identify sepsis in the prehospital setting: a validation study.* CMAJ, 2020. **192**(10): p. E230-E239.
19. Park, J., et al., *Emergency medical service personnel need to improve knowledge and attitude regarding prehospital sepsis care.* Clin Exp Emerg Med, 2017. **4**(1): p. 48-55.
20. Sjosten, O., et al., *The prehospital assessment of patients with a final hospital diagnosis of sepsis: Results of an observational study.* Australas Emerg Care, 2019. **22**(3): p. 187-192.
21. Smyth, M.A., S.J. Brace-McDonnell, and G.D. Perkins, *Identification of adults with sepsis in the prehospital environment: a systematic review.* BMJ Open, 2016. **6**(8): p. e011218.
22. Rosenqvist, M., et al., *Improved Outcomes After Regional Implementation of Sepsis Alert: A Novel Triage Model.* Crit Care Med, 2020. **48**(4): p. 484-490.

For peer review only

23. Latten, G.H.P., et al., *Characteristics of the prehospital phase of adult emergency department patients with an infection: A prospective pilot study.* PLoS One, 2019. **14**(2): p. e0212181.

24. Seymour, C.W., et al., *Delays From First Medical Contact to Antibiotic Administration for Sepsis.* Crit Care Med, 2017. **45**(5): p. 759-765.

25. Widgren, B.R. and M. Jourak, *Medical Emergency Triage and Treatment System (METTS): a new protocol in primary triage and secondary priority decision in emergency medicine.* J Emerg Med, 2011. **40**(6): p. 623-8.

26. Widgren, B.R., M. Jourak, and A. Martinius, *[New accurate triage method. METTS-A yields basis for priority level decisions].* Lakartidningen, 2008. **105**(4): p. 201-4.

27. Vincent, J.L., et al., *The SOFA (Sepsis-related Organ Failure Assessment) score to describe organ dysfunction/failure. On behalf of the Working Group on Sepsis-Related Problems of the European Society of Intensive Care Medicine.* Intensive Care Med, 1996. **22**(7): p. 707-10.

28. *Royal College of Physicians. National Early Warning Score (NEWS) 2: Standardising the assessment of acute-illness severity in the NHS. Updated report of a working party. London: RCP, 2017.*

29. Ammerlaan, H.S., et al., *Secular trends in nosocomial bloodstream infections: antibiotic-resistant bacteria increase the total burden of infection.* Clin Infect Dis, 2013. **56**(6): p. 798-805.

30. Quan, H., et al., *Updating and validating the Charlson comorbidity index and score for risk adjustment in hospital discharge abstracts using data from 6 countries.* Am J Epidemiol, 2011. **173**(6): p. 676-82.

31. Quan, H., et al., *Coding algorithms for defining comorbidities in ICD-9-CM and ICD-10 administrative data.* Med Care, 2005. **43**(11): p. 1130-9.

32. Sundararajan, V., et al., *New ICD-10 version of the Charlson comorbidity index predicted in-hospital mortality.* J Clin Epidemiol, 2004. **57**(12): p. 1288-94.

33. Singer, M., et al., *The Third International Consensus Definitions for Sepsis and Septic Shock (Sepsis-3).* JAMA, 2016. **315**(8): p. 801-10.

34. *Empirical antibiotic treatment in severe infections.* 2021-08-26]; Available from: https://www.sls.se/globalassets/raf/ovriga-dokument/rodakortet-uppdaterat-150528.pdf.

35. *Swedish Strama antibiotic stewardship programme. Available; https://www.folkhalsomyndigheten.se/the-public-health-agency-of-sweden/communicable-disease-control/antibiotics-and-antimicrobial-resistance/swedish-work-on-containment-of-antibiotic-resistance/ Accessed 2021-03-10.*

36. *The European Committee on Antimicrobial Susceptibility Testing. Breakpoint tables for interpretation of MICs and zone diameters. Version 9.0, 2019. http://www.eucast.org.*

37. Andersson, M., et al., *Delay of appropriate antibiotic treatment is associated with high mortality in patients with community-onset sepsis in a Swedish setting.* Eur J Clin Microbiol Infect Dis, 2019.

38. Henriksen, D.P., et al., *Incidence rate of community-acquired sepsis among hospitalized acute medical patients-a population-based survey.* Crit Care Med, 2015. **43**(1): p. 13-21.

39. Jacobsson, G., E. Gustafsson, and R. Andersson, *Outcome for invasive Staphylococcus aureus infections.* Eur J Clin Microbiol Infect Dis, 2008. **27**(9): p. 839-48.

40. Melzer, M. and C. Welch, *Thirty-day mortality in UK patients with community-onset and hospital-acquired meticillin-susceptible Staphylococcus aureus bacteraemia.* J Hosp Infect, 2013. **84**(2): p. 143-50.

41. Whiles, B.B., A.S. Deis, and S.Q. Simpson, *Increased Time to Initial Antimicrobial Administration Is Associated With Progression to Septic Shock in Severe Sepsis Patients.* Crit Care Med, 2017. **45**(4): p. 623-629.

42. Seymour, C.W., et al., *Time to Treatment and Mortality during Mandated Emergency Care for Sepsis.* N Engl J Med, 2017. **376**(23): p. 2235-2244.

For peer review only

43. Lane, D., et al., *Prehospital management and identification of sepsis by emergency medical services: a systematic review.* Emerg Med J, 2016. **33**(6): p. 408-13.
44. Velasco, E., et al., *Bloodstream infection surveillance in a cancer centre: a prospective look at clinical microbiology aspects.* Clin Microbiol Infect, 2004. **10**(6): p. 542-9.
45. Corfield, A.R., et al., *Utility of a single early warning score in patients with sepsis in the emergency department.* Emerg Med J, 2014. **31**(6): p. 482-7.
46. Churpek, M.M., et al., *Quick Sepsis-related Organ Failure Assessment, Systemic Inflammatory Response Syndrome, and Early Warning Scores for Detecting Clinical Deterioration in Infected Patients outside the Intensive Care Unit.* Am J Respir Crit Care Med, 2017. **195**(7): p. 906-911.
47. Gerry, S., et al., *Early warning scores for detecting deterioration in adult hospital patients: systematic review and critical appraisal of methodology.* BMJ, 2020. **369**: p. m1501.
48. Rhodes, A., et al., *Surviving Sepsis Campaign: International Guidelines for Management of Sepsis and Septic Shock: 2016.* Intensive Care Med, 2017. **43**(3): p. 304-377.

For peer review only

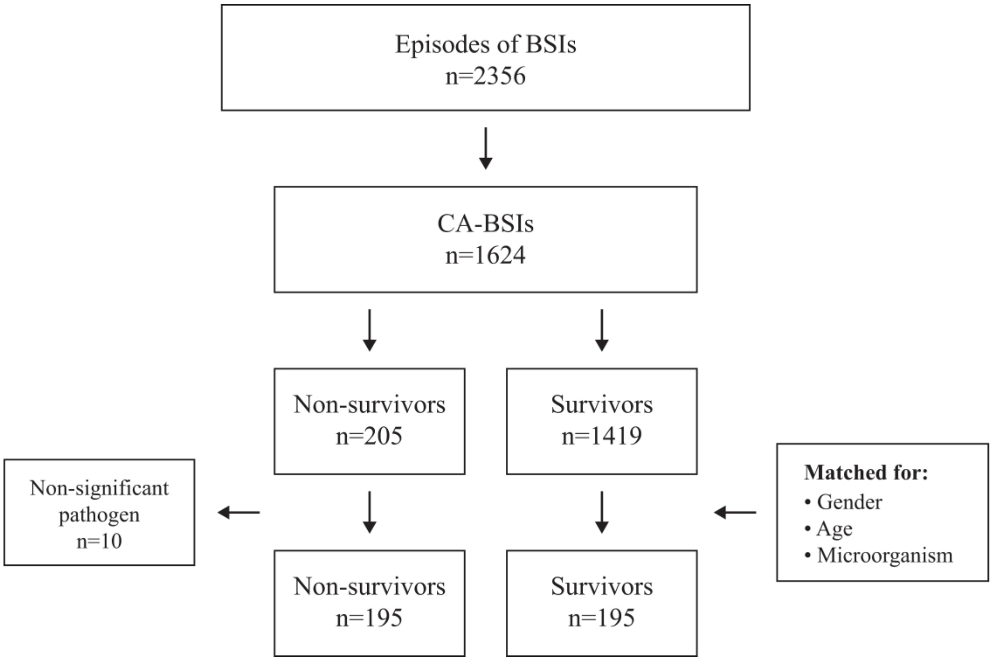

Fig 1. A total of 2,356 BSI episodes were identified, of which 1,624 were CA-BSIs, 195 non-survivors met the inclusion criteria and were matched with 195 survivors for age (+/- 10 years), gender, and microorganisms.

334x218mm (600 x 600 DPI)

Supplementary Table 1. Charlson Comorbidity Index

Comorbid Condition	ICD-9-CM Diagnosis Codes	ICD-10-CA Diagnosis Codes	Weight	Updated Weight
**Myocardial Infarction**	410, 412	I21, I22, I25.2	1	0
**Congestive Heart Failure**	398.91, 402.01, 402.11, 402.91, 404.01, 404.03, 404.11, 404.13, 404.91, 404.93, 425.4–425.9, 428 (hosp), 398, 402, 425, 428 (med)	I09.9, I11.0, I13.0, I13.2, I25.5, I42.0, I42.5–I42.9,I43, I50, P29.0	1	2
**Peripheral Vascular Disease**	093.0, 437.3, 440, 441, 443.1–443.9, 447.1, 557.1, 557.9, V43.3 (hosp) 440, 441, 443, 447, 557 (med)	I70, I71, I73.1, I73.8, I73.9, I77.1, I79.0, I79.2, K55.1, K55.8, K55.9, Z95.8, Z95.9	1	0
**Cerebrovascular Disease**	362.34, 430–438 (hosp) 430–438 (med)	G45, G46, H34.0, I60–I69	1	0
**Dementia**	290, 294.1, 331.2 (hosp) 290, 294, 331 (med)	F00–F03, F05.1, G30, G31.1	1	2
**Chronic Pulmonary Disease**	416.8, 416.9, 490–505, 506.4, 508.1, 508.8 (hosp), 416, 490–496, 500–505 (med)	I27.8, I27.9,J40–J47, J60–J67 J68.4, J70.1, J70.3	1	1
**Connective Tissue Disease-Rheumatic Disease**	446.5, 710.0–710.4, 714.0–714.2, 714.8, 725 (hosp), 446, 710, 714, 725 (med)	M05, M06, M31.5, M32–M34, M35.1, M35.3, M36.0	1	1
**Peptic Ulcer**	531–534	K25–K28	1	0
**Mild Liver Disease**	070.22, 070.23, 070.32, 070.33, 070.44, 070.54, 070.6, 070.9, 570, 571, 573.3, 573.4, 573.8, 573.9, V42.7 (hosp) 070, 570, 571, 573 (med)	B18, K70.0–K70.3, K70.9, K71.3–K71.5, K71.7, K73, K74, K76.0, K76.2–K76.4, K76.8, K76.9, Z94.4	1	2
**Diabetes without Chronic Complications**	250.0–250.3, 250.8, 250.9 (hosp) 250 (med)	E10.0, E10.1, E10.6, E10.8, E10.9, E11.0, E11.1, E11.6, E11.8, E11.9, E12.0, E12.1, E12.6, E12.8, E12.9, E13.0, E13.1, E13.6, E13.8, E13.9, E14.0, E14.1, E14.6, E14.8, E14.9	1	0
**Diabetes with Chronic Complications**	250.4–250.7 (med n/a)	E10.2–E10.5, E10.7, E11.2–E11.5, E11.7, E12.2–E12.5, E12.7, E13.2–E13.5, E13.7, E14.2–E14.5, E14.7	2	1
**Paraplegia or Hemiplegia**	334.1, 342, 343, 344.0–344.6, 344.9 (hosp), 334, 342–344 (med)	G04.1, G11.4, G80.1, G80.2, G81, G82, G83.0–G83.4, G83.9	2	2

For peer review only

**Renal Disease**	403.01, 403.11, 403.91, 404.02, 404.03, 404.12, 404.13, 404.92, 404.93, 582, 583.0–583.7, 585, 586, 588.0. V42.0, V45.1, V56 (hosp), 403, 582, 583, 585, 586, 588, V56 (med)	I12.0, I13.1, N03.2–N03.7, N052–N05.7, N18, N19, N25.0, Z49.0–Z49.2, Z94.0, Z99.2	2	1
**Cancer ***	140–172, 174–195.8, 200–208, 238.6 (hosp), 140–172, 174–195, 200–208, 238 (med)	C00–C26, C30–C34, C37–C41, C43, C45–C58, C60–C76, C81–C85, C88, C90–C97	2	2
**Moderate or Severe Liver Disease**	456.0–456.2, 572.2–572.4, 572.8 (hosp), 456, 572 (med)	I85.0, I85.9, I86.4, I98.2, K70.4, K71.1, K72.1, K72.9, K76.5–K76.7,	3	4
**Metastatic Carcinoma****	196–199	C77–C80	6	6
**HIV/AIDS**	042–044	B20–B22, B24	6	4

*Abbreviations: AIDS, acquired immunodeficiency syndrome; HIV, human immunodeficiency virus. * Including lymphoma and leukemia, except malignant neoplasm of skin ** or Metastatic solid tumour.*

For peer review only

Supplementary Table 2. Distribution of comorbidities

	**Non-survivors n=195**	**Survivors n=195**	**p-value**
**Comorbidity (%)**			
Myocardial infarction	31 (16)	41 (21)	0.19
Congestive heart failure	56 (29)	50 (26)	0.50
Peripheral vascular disease	14 (7)	16 (8)	0.70
Cerebrovascular disease	30 (15)	28 (14)	0.78
Dementia	15 (8)	11 (6)	0.42
Chronic pulmonary disease	35 (18)	33 (17)	0.79
Connective Tissue Disease-Rheumatic Disease	13 (7)	22 (11)	0.11
Peptic ulcer disease	3 (2)	3 (2)	>0.99
Mild liver disease	8 (4)	9 (5)	0.80
Diabetes without chronic complications	43 (22)	62 (32)	*0.03*
Diabetes with chronic complications	8 (4)	9 (5)	0.80
Paraplegia and hemiplegia	13 (7)	11 (6)	0.67
Renal disease	34 (17)	25 (13)	0.20
Cancer	67 (34)	44 (23)	*0.01*
Moderate or severe liver disease	9 (5)	3 (2)	0.08
Metastatic carcinoma	28 (14)	14 (7)	*0.02*
HIV/AIDS	0	0	-
Charlson (update weight) (SD)	3.2 (3.0)	2.3 (2.4)	*0.01*

Data are presented as no. (%) or mean (SD). Pearson chi2, Fisher's exact test or T-test, as appropriate.

For peer review only

Supplementary Table 3. Distribution of primary pathogen*

**Gram negative**	**Non-survivors n=195**	**Survivors n=195**	**%**
**Enterobacterales**			
Escherichia coli	41	41	21
Klebsiella pneumoniae	14	14	7
Klebsiella oxytoca	3	3	2
Enterobacter cloacae	2	2	1
Proteus Mirabilis	4	4	2
Haemophilus influenzae	4	4	2
**Other than Enterobacterales**			
Pseudomonas aeruginosa	7	7	4
**Others**	7	7	4
**Gram positives**			
**Enterococci**			
Enterococcus faecalis	6	6	3
Enterococcus faecium	5	5	3
**Staphylococci**			
Staphylococcus aureus	52	52	27
Staphylococcus spp	5	5	3
**Streptococci**			
Streptococcus pneumoniae	16	16	8
Streptococcus pyogenes (A)	1	1	1
Streptococcus agalactiae (B)	4	4	2
Streptococcus group C,G	5	5	3
Streptococcus spp	11	11	6
***Candida* spp**	4	4	2
**Others**	4	4	2
**Polymicrobial-BSI**	9	8	4

**Primary pathogen reported in the table. In Polymicrobial-BSI, an expert group (physicians specialised in infectious diseases) evaluated which pathogen was primary.*

For peer review only

Supplementary Table 4. Hospital data - Triage at hospital (RETTS and NEWS 2) and contact cause at hospital

	**Non-survivors n=195**	**Survivors n=195**	**p-value**
**Vital signs (SD)***			
Body temp (BT)	37.6 (1.3) n189	38.2 (1.1) n194	*<0.01*
Systolic blood pressure (SBP)	112 (28) n190	131 (28) n193	*<0.01*
Diastolic blood pressure (DBP)	65 (18) n188	72 (17) n192	*<0.01*
Pulse rate (HR)	102 (26) n190	97 (22) n192	*0.04*
Respiratory rate (RR)	27 (10) n184	23 (7) n189	*<0.01*
Blood O_2 saturation (SPO_2)	92 (9) n187	95 (4) n192	*<0.01*
PaO_2/FiO_2	42 (21) n183	51 (20) n187	*<0.01*
Reaction Level Scale (RLS)	1.4 (0.9) n187	1.1 (0.46) n192	*<0.01*
**Laboratory median (Q1-Q3)**			
C-reactive protein (mg/L)	180 (87-304) n195	138 (50-225) n192	*<0.01*
White blood cell count (x10^9/L)	13 (8-19) n195	13(10-17) n191	0.42
Platelet count (x10^9/L)	194 (130-292) n195	212 (156-280) n191	0.09
Creatinine (µmol/L)	143 (94-216) n191	98 (74-139) n190	*<0.01*
eGFR MDRD (mL/min/1.73 m^2)	36 (22-58) n186	58 (35-74) n186	*<0.01*
Albumin (g/L)	23 (20-28) n109	29 (25-32) n100	*<0.01*
B-Erythrocytes (x10^{12}/L)	4 (3-4) n195	4 (4-5) n191	*0.04*
B-MCV (fL)	95 (91-101) n195	93 (90-97) n191	*<0.01*
Haemoglobin (g/L)	116 (101-136) n195	121 (109-138) n191	*0.02*
Procalcitonin (µg/L)	10 (2-54) n34	4 (1-31) n26	0.33
**Primary focus of infection (%)**			
Respiratory tract	39 (20)	46 (24)	0.39
Urinary tract	42 (22)	50 (26)	0.34
Skin and soft tissue	19 (10)	20 (10)	0.86
Abdomen	12 (6)	18 (9)	0.25
CNS	-	2 (1)	0.50
Indwelling catheter	1 (1)	0	>0.99
Endocarditis	1 (1)	1 (1)	>0.99
Unknown	46 (24)	43 (22)	0.72
Missing data	35 (18)	15 (8)	
**Triage**			
RETTS Red (%)	76 (46) n164	62 (35) n177	*0.03*
RETTS Orange (%)	51 (31) n164	71 (40) n177	0.08
RETTS Yellow (%)	32 (20) n164	42 (24) n177	0.35
RETTS Green (%)	5 (3) n164	2 (1) n177	0.27
RETTS Red plus Orange (%)	127 (77) n164	133 (75) n177	0.62
NEWS 2 score (SD)	7.1 (4.0) n163	5.0 (3.4) n181	*<0.01*

For peer review only

NEWS 2 score ≥5 (%)	114 (70) n163	89 (49) n181	*<0.01*
**Reason for first hospital contact (%)**			
Fever	86 (44)	119 (61)	*<0.01*
Chills	26 (13)	50 (26)	*0.02*
“Found on the floor”	18 (9)	5 (3)	*0.01*
Gastrointestinal symptoms	32 (16)	32 (16)	>0.99
Cough	8 (4)	15 (8)	0.13
Dyspnoea/breathing difficulties	60 (31)	46 (24)	0.11
Urinary tract symptoms	13 (7)	25 (13)	*0.04*
Rapid deterioration in general condition	81 (42)	48 (25)	*<0.01*
Fatigue	14 (7)	17 (9)	0.57

*Data are presented as no. (%) or mean (SD). Pearson chi2, Fisher’s exact test or T-test, as appropriate. Laboratory data are calculated with median, interquartile 25th to 75th percentile range (Q1-Q3) and Mann–Whitney U. * Vital signs first registered at admission. RETTS: Red = life-threatening condition, Orange = potentially life-threatening. Yellow/Green = acute process can wait.*

For peer review only

Supplementary Table 5. Early hospital care - Time for ambulance transport and time to empirical antibiotic treatment based on triage score (RETTS)

**Early hospital care**	**Non-survivors**	**Survivors**	**p-value**
Paramedic alarm times, hours, median (Q1-Q3)	0,72 (0,57-0,95), n137	0,73 (0,58-0,98), n131	0.40
Time to empirical antibiotic treatment based on triage score (RETTS), hours, median (Q1-Q3):			
Red (triage 1)	1,52 (0,67-3,18), n68	1,62 (0,90-2,98), n62	0.50
Orange (triage 2)	2,63 (1,72-5,15), n50	3,07 (1,63-6,13), n69	0.67
Yellow (triage 3)	4,88 (3,72-7,30), n32	5,28 (2,75-9,97), n38)	0.80
Green (triage 4)*	9,67 (6,26), n5	11,23 (8,60), n2	0.79

*Data are presented as hours and median (Q1-Q3) or * mean and SD. Mann-Whitney U test or T-test are used, as appropriate. RETTS: Red = life-threatening condition, Orange = potentially life-threatening. Yellow/Green = acute process can wait.*

For peer review only

Supplementary Table 6. Distribution of Empirical antibiotics used

	Non-survivors (n186)	Survivors (n192)	p-value
**Classes of antibiotics:**			
Aminoglycosides	15 (8)	9 (5)	0.18
Penicillins + beta-lactamase inhibitors	53 (28)	43 (22)	0.18
Carbapenems	23 (12)	8 (4)	<0.01
Cephalosporins	90 (48)	121 (63)	<0.01
Quinolones	3 (2)	1 (1)	0.37
Trimethoprim-sulfamethoxazole	0 (0)	1 (1)	>0.99
Glycopeptides	1 (1)	1 (1)	>0.99
Lincosamides	9 (5)	5 (3)	0.25
Macrolides	3 (2)	2 (1)	0.68
Nitroimidazoles	3 (2)	2 (1)	0.68
Penicillins	15 (8)	20 (10)	0.53
Antifungals	1 (1)	2 (1)	>0.99
Others	1 (1)	0 (0)	0.49
**Monotherapy treatment**	156 (84)	173 (90)	0.08
**Combination therapy treatment**	31 (17)	21 (11)	0.11

Data are presented as no. (%). Pearson chi2 or Fisher's exact test as appropriate. Combination therapy treatment was defined as more than one agent given within 4 hours in order to broaden the antimicrobial spectrum. Seven patients received oral antibiotic treatment, 4 non-survivors and 3 survivors.

For peer review only

STROBE Statement—Checklist of items that should be included in reports of case-control studies

	Item No	Page No
**Titel and abstract**	1	1
**Introduction**		
Background	2	3-4
Objectives	3	4
**Methods**		
Study design	4	4
Setting	5	4
Participants	6	4-5
Variables	7	4-7
Data sources	8	4-6
Bias	9	N/A
Study size	10	4-5
Quantitative variables	11	8
Statistical methods	12	8
**Results**		
Participants	13	9, Flow-chart
Descriptive data	14	9, table 1,2 and supplementary files
Outcome data	15	9-11, Table 1, 2
Main results	16	11-12
Other analyses	17	N/A
**Discussion**		
Key results	18	12
Limitations	19	15-16
Interpretation	20	12-15, 16
Generalisability	21	12-15, 16
**Other information**		
Funding	22	16

For peer review only
